# Pollination by nocturnal Lepidoptera, and the effects of light pollution: a review

**DOI:** 10.1111/een.12174

**Published:** 2014-12-13

**Authors:** Callum J MacGregor, Michael J O Pocock, Richard Fox, Darren M Evans

**Affiliations:** 1 School of Biological, Biomedical and Environmental Sciences, University of HullHull, U.K.; 2 Centre for Ecology & HydrologyWallingford, U.K.; 3 Butterfly ConservationWareham, U.K.

**Keywords:** Agro-ecosystems, artificial night lighting, ecological networks, ecosystem services, flowering plants, food-webs, moths, population declines

## Abstract

1. Moths (Lepidoptera) are the major nocturnal pollinators of flowers. However, their importance and contribution to the provision of pollination ecosystem services may have been under-appreciated. Evidence was identified that moths are important pollinators of a diverse range of plant species in diverse ecosystems across the world.

2. Moth populations are known to be undergoing significant declines in several European countries. Among the potential drivers of this decline is increasing light pollution. The known and possible effects of artificial night lighting upon moths were reviewed, and suggest how artificial night lighting might in turn affect the provision of pollination by moths. The need for studies of the effects of artificial night lighting upon whole communities of moths was highlighted.

3. An ecological network approach is one valuable method to consider the effects of artificial night lighting upon the provision of pollination by moths, as it provides useful insights into ecosystem functioning and stability, and may help elucidate the indirect effects of artificial light upon communities of moths and the plants they pollinate.

4. It was concluded that nocturnal pollination is an ecosystem process that may potentially be disrupted by increasing light pollution, although the nature of this disruption remains to be tested.

## Introduction

Pollinating insects have been undergoing significant declines for several decades in many parts of the world (Williams, 1982; Potts *et al.,* 2010; Carvalheiro *et al.,* 2013). This is of concern because pollination represents a critical ecosystem service (Costanza *et al.,* 1997; Ollerton *et al.,* 2011; Garibaldi *et al.,* 2013), and declines in pollinators have been linked with declines in the plants that they interact with (Biesmeijer *et al.,* 2006; Pauw, 2007; Potts *et al.,* 2010). However, most studies to date have focused on diurnal pollinating insects, largely ignoring nocturnal insects, many of which have also undergone significant declines. In Great Britain, two-thirds of widespread larger moth species populations declined over a 40-year period (Fox *et al.,* 2013), with probable detrimental cascading effects on ecosystem functioning: the nature of these is considered a priority, policy-relevant question (Sutherland *et al.,* 2006). Recent work suggests that nocturnal moths (Lepidoptera) may perform an important, although often overlooked, functional role as plant pollinators (Philipp *et al.,* 2006; Devoto *et al.,* 2011; LeCroy *et al.,* 2013), but little is known about the scale and importance of nocturnal pollination services. Here, we review the scientific literature for evidence of the importance of nocturnal Lepidoptera (moths) as plant pollinators.

Nocturnal insect pollinators, including moths, face many of the same threats as diurnal pollinators, including habitat fragmentation, climate change, and agrochemical use (Fox *et al.,* 2014). They may also be affected by increasing light pollution (Hölker *et al.,* 2010a), but the effects of artificial night lighting on nocturnal pollinator communities have not yet been established. We examine how the known effects of artificial light upon moths may potentially affect pollination processes. We also consider how recent advances in network ecology can be used to examine the impacts of light pollution on moth communities and their interactions with plants.

## Nocturnal pollination

**Fig 1:**
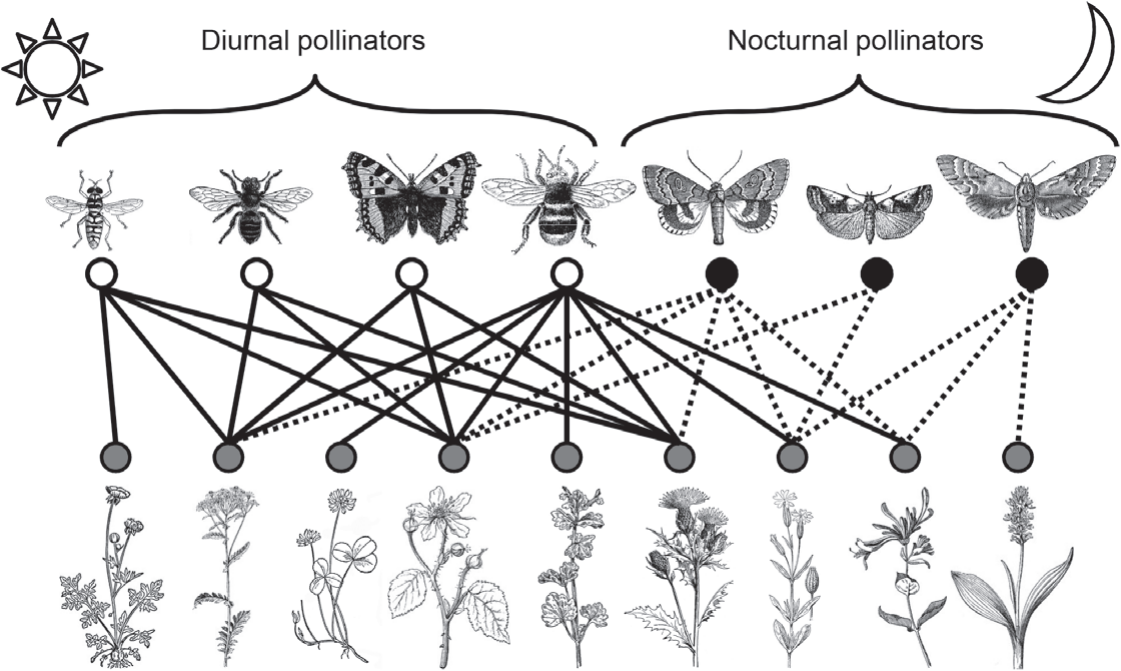
An illustrative temperate grassland network combining diurnal and nocturnal pollination. Combined networks may reveal the extent of redundancy and complementarity of pollination interactions in ecosystems. Some apparently specialist plants in diurnal networks may be generalist with nocturnal visitors included. Thus, nocturnal visitors may provide redundancy to plants pollinated by diurnal visitors, and vice versa. Nocturnal interactions are derived from Table S1.2, Appendix S2 and diurnal interactions from Pocock *et al.* (2012). Nodes represent species: white = diurnal insects, black = nocturnal insects, grey = plants. Pollinators (from left): hoverfly (Diptera), leaf-cutter bee (Hymenoptera), butterfly (Lepidoptera), bumblebee (Hymenoptera), noctuid moth, pyralid moth, sphingid moth (all Lepidoptera); plants (from left): *Ranunculus* sp. (Ranunculaceae), *Jacobaea vulgaris* (Asteraceae), *Trifolium* sp. (Fabaceae), *Rubus* sp. (Rosaceae), *Lamium* sp. (Lamiaceae), *Cirsium* sp. (Asteraceae), *Silene latifolia* (Caryophyllaceae), *Lonicera* sp. (Caprifoliaceae), *Gymnadenia conopsea* (Orchidaceae). Links represent hypothetical pollination interactions: solid = diurnal, dashed = nocturnal. Drawings of pollinators and plants are for illustration only and may not precisely represent the named plant or animal. Drawings are used under license from ClipArt ETC (see Appendix S1 for full acknowledgements).

The experimental methods used in the majority of field studies of plant–pollinator interactions involve observations of insect visitors to flowers. Such observations almost always take place during daylight hours (e.g. Forup *et al.,* 2008; Bosch *et al.,* 2009; Popic *et al.,* 2013), because conducting surveys in the dark is difficult (Martinell *et al.,* 2010). However, to fully understand plant–pollinator networks, we must also understand the role played by nocturnal pollinators (Fig. 1). In addition to some bats (Chiroptera), beetles (Coleoptera), and flies (Diptera), moths are important nocturnal pollinators (Willmer, 2011); in particular, nectarivorous species from the families Sphingidae, Noctuidae, and Geometridae (Winfree *et al.,* 2011) and probably also the newly defined Erebidae (LeCroy *et al.,* 2013).

To determine the importance of moths as providers of nocturnal pollination services, and which plants are pollinated, we searched ISI Web of Knowledge for papers containing the terms ‘moth’ and ‘pollinat*’ (30 January 2014) and searched the bibliography of each relevant publication for further citations. Any paper demonstrating the existence of a moth–plant pollination interaction or providing evidence for such an interaction was considered relevant and included in the review. Levels of evidence supporting pollination interactions varied from observed flower visitation alone to proven dependence of the flower on moths for pollination (Table 1). Eight studies only inferred moth pollination from floral characteristics and did not present further evidence. While a high proportion of flower visitors at any particular flower species may not be effective pollinators (King *et al.,* 2013), flower visitation or pollen transfer by insects is frequently used as a proxy for insect-pollination. Therefore, for simplicity, we hereafter use the terms ‘pollination’ and ‘pollinator’ where there was reasonable evidence that moths acted as pollinators, although we note that in many cases pollination was not strictly proven. Using this method, we identified 168 studies from between 1971 and 2013 detailing examples of nocturnal moths involved in pollination (this search was comprehensive, but we recognise that some additional published examples may exist).

**Table 1:** Types of evidence for moth pollination given by studies reviewed (see Table S1.2, Appendix S2)

Evidence	Types of evidence	No. studies
Only flower visitation recorded	VF, VO, VR, VT	52
Flower visitation and moths observed making contact with floral reproductive organs	C + (VF, VO, VR, VT)	11
Only pollen found on moths	P	15
Flower visitation recorded and pollen found on moths	P + (VF, VO, VR, VT)	49
Flower visitation recorded with other additional evidence	(VF, VO, VR, VT) + X	9
Pollen found on moths with other additional evidence	P + X	2
Flower visitation and pollen found on moths with other additional evidence	P + (VF, VO, VR, VT) + X	8
Other	X	4
Only inferred from floral syndrome	I	8
Unspecified/unknown	U	5

In column 2: C = contact with anthers and/or stigmas observed, D = pollen deposited on stigmas and/or removed from anthers, E = plants pollinated when experimentally exposed only to visits by moths, I = inferred from pollination syndrome, P = pollen present on captured moths, S = moth scales or hairs present on stigmas, VF = flower visitation determined by fluorescent markers transferred by visiting moths, VO = flower visitation determined by observations, VR = flower visitation determined by video recordings, VT = flower visitation determined by flower-visitor trapping, U = unspecified/unknown; X = any combination of C, D, E, and S.

Fourteen of these studies examined complete pollinator communities, finding moths to be of general importance to pollination in a variety of ecosystems (Table S1.1, Appendix S2), including tropical rainforest and savannah, temperate coniferous forest and meadow, and oceanic islands, and including examples from all continents except Antarctica. In several studies, moths were considered to be second in importance only to bees, in terms of pollination provision (Bawa *et al.,* 1985; Kato & Kawakita, 2004; Ramirez, 2004; Chamorro *et al.,* 2012).

Moth pollination was important for a wide range of plant species. We found representatives of 75 different plant families (Table 2), including 289 species and some wider taxa, reported to be partially or exclusively pollinated by moths (Table S1.2, Appendix S2) of 21 families (Table S3, Appendix S2). The majority of plants were angiosperms; the one exception was the gymnosperm *Gnetum gnemon* Linne var. *tenerum* Markgraf (Gnetaceae), reportedly pollinated by moths of Geometridae and Pyralidae (Kato *et al.,* 1995). Many species within the angiosperms were dicotyledons, especially from the orders Caryophyllales, Ericales, Gentianales, and Lamiales, but moth-pollinated plants in the monocotyledons included many in the order Asparagales (including Orchidaceae, Amaryllidaceae, Asparagaceae, and others), and the economically important species *Elaeis guineensis* Jacq. oil palm (Arecaceae), visited by large numbers of moths in the genus *Pyroderces* (Cosmopterigidae; Syed, 1979). These observed patterns may be a function of both real effects and bias in recorder effort, so we treat them with caution.

**Table 2:** Studies of moth-pollinated plants by family (see Table S1.2, Appendix S2)

Plant family	No. known moth-pollinated species or wider taxa	Known pollinating moth families	Plant family	No. known moth-pollinated species or wider taxa	Known pollinating moth families
Adoxaceae	1	N	Liliaceae	4	G, N, P, S
Amaranthaceae	1	—	Linaceae	1	—
Amaryllidaceae	10	E, N, S	Loasaceae	1	S
Anacardiaceae	1	—	Loganiaceae	2	—
Apiaceae	1	—	Malvaceae	2	Ct, E, G, N, P, Se, S, U
Apocynaceae	20	E, G, N, P, S, T	Meliaceae	1	S
Arecaceae	1	C	Myrtaceae	2	Ct, S
Asparagaceae	7	N, Pr, S	Nepenthaceae	1	—
Asteraceae	13	G, N, P, S	Nyctaginaceae	5	N, S
Balsaminaceae	2	S	Oleaceae	3	S
Bignoniaceae	3	E, G, L, N, S	Onagraceae	8	E, G, N, P, S
Boraginaceae	4	N, P, S	Orchidaceae	45	G, N, Pr, Pt, P, Se, S, T
Brassicaceae	3	S	Orobanchaceae	2	S
Cactaceae	7	G, N, P, Sa, S	Passifloraceae	2	S
Capparaceae	1	P	Phrymaceae	1	S
Caprifoliaceae	3	N, S	Phyllanthaceae	10	Ge, Gr
Caricaceae	1	—	Plantaginaceae	1	—
Caryocaraceae	1	S	Polemoniaceae	1	S
Caryophyllaceae	12	Cr, G, N, P, S	Polygonaceae	1	—
Cleomaceae	1	S	Primulaceae	2	—
Convulvulaceae	4	S	Proteaceae	2	S
Crassulaceae	1	G	Ranunculaceae	5	S
Cucurbitaceae	1	N, S	Rhamnaceae	1	—
Dipterocarpaceae	2	G, N, S	Rosaceae	2	—
Ebenaceae	1	—	Rubiaceae	16	Ct, N, S
Ericaceae	4	G, N, P, S	Rutaceae	1	G
Escalloniaceae	1	G	Santalaceae	2	—
Euphorbiaceae	4	S	Sapotaceae	2	—
Fabaceae	12	E, G, N, P, S, U	Saxifragaceae	3	Pr
Geraniaceae	1	—	Scrophulariaceae	2	G, N, P, T
Gesneriaceae	1	—	Solanaceae	6	S
Gnetaceae	1	G, P	Thymelaeaceae	8	E, G, L, N, No, P, Th
Hyacinthaceae	1	N	Urticaceae	1	—
Hypericaceae	1	N	Verbenaceae	3	P, S
Iridaceae	3	G, N, S	Violaceae	1	S
Lamiaceae	2	S	Vochysiaceae	5	S
Lecythidaceae	1	Gl	Winteraceae	2	M
Lentibulariaceae	1	N, P, S, U	—	—	—

In column 2, ‘known’ moth-pollinated taxa are those identified in this review as having evidence of being moth-pollinated; ‘wider taxa’ includes any named group at a hierarchical level above species and below family. In column 3: C = Cosmopterigidae, Cr = Crambidae, Ct = Ctenuchidae, E = Erebidae, Ge = Gelechiidae, G = Geometridae, Gl = Glyphipterigidae, Gr = Gracillariidae, L = Lasiocampidae, M = Micropterigidae, N = Noctuidae, No = Nolidae, Pr = Prodoxidae, Pt = Pterophoridae, P = Pyralidae, Sa = Saturniidae, Se = Sesiidae, S = Sphingidae, Th = Thyrididae, T = Tortricidae, U = Uranidae.

Traditionally, pollination by moths has been subdivided into two ‘pollination syndromes’ (Willmer, 2011): sphingophily (pollination by hovering moths of the Sphingidae) and phalaenophily (pollination by settling moths of other families). The best-known examples of moth pollination are of sphingophilous plants (e.g. Wasserthal, 1997). To examine if this has led to a bias towards sphingophily in studies of moth pollination, we categorised all studies in Table S1.2, Appendix S2 according to whether they made any explicit or implicit prediction of sphingophily. In general, we did not find evidence of bias towards sphingophily leading to other pollination interactions being overlooked. Fifty-six studies (35% of those reviewed) made a prediction of sphingophily. Of these, 53 (95%) found Sphingidae and 18 (32%) found non-sphingid moths to be pollinators, even although the experimental methods in all but two studies were sufficient to detect both sphingid and non-sphingid pollinators. From the 103 studies not predicting sphingophily, 82 (80%) found non-sphingid moths and 50 (49%) found Sphingidae to be pollinators; the experimental methods in all but nine were sufficient to detect both sphingid and non-sphingid pollinators (Table S2, Appendix S2).

Moths primarily visit flowers to obtain nectar, which is an energy-rich food source and the main adult food source in the majority of moth species that feed as adults (Willmer, 2011). Several studies have also documented moths acting as pollinating seed parasites (Table S1.3, Appendix S2). In these specialised interactions, moths both pollinate and lay eggs in flowers, so providing a food supply for their larvae, which feed on developing seedheads.

Pollination by moths may be an advantageous strategy for plants in some examples. Several studies evaluate aspects of pollination in generalist plants pollinated both by moths (both Sphingidae and other families) and diurnal pollinators; for example, *Lonicera japonica* Thunb. (Caprifoliaceae; Miyake & Yahara, 1998), *Asclepias* spp. (Apocynaceae; Bertin & Willson, 1980; Morse & Fritz, 1983; Jennersten & Morse, 1991) and *Silene* spp. (Caryophyllaceae; Young, 2002; Barthelmess *et al.,* 2006). Compared with diurnal pollinators, the moths in these examples provided benefits including: greater interpopulation gene flow, shown by movement of genetic markers between experimental populations of plants (Barthelmess *et al.,* 2006); longer-distance dispersal of dye-marked pollen (Miyake & Yahara, 1998; Young, 2002); higher quality pollination, causing equal or greater seed set in spite of transferring fewer pollinia (Bertin & Willson, 1980; Jennersten & Morse, 1991; but see Morse & Fritz, 1983); and more efficient pollination, having a lower ratio of pollen removed to pollen deposited after visits by single pollinators (Miyake & Yahara, 1998). In the latter example, moths visiting *L. japonica* were thought to be more efficient pollinators than bees because the latter actively collect pollen to provision their larvae, and so must remove substantially more pollen than moths for the same level of pollen deposition to occur (Miyake & Yahara, 1999). As a result, moth-pollinated plants could perhaps invest fewer resources into producing pollen without compromising reproductive success (Cruden, 1973); however, analysis of pollen–ovule ratios for diurnally and nocturnally pollinated members of Caryophyllaceae does not support this (Jürgens *et al.,* 2002).

The literature, therefore, contains numerous examples of moths serving as pollinators which, in many cases, are of considerable importance to individual species and to communities. A diverse selection of plant taxa in an equally wide range of ecosystems benefit from pollination by moths. It is important to consider how environmental change may threaten this ecosystem service.

## Artificial light as a driver of environmental change

There are many drivers of environmental change, but artificial night lighting is one which is uniquely important for nocturnal organisms, through direct interaction with a light source such as a streetlamp, increased background illumination at night, and altered perception of photoperiod (Hölker *et al.,* 2010b; Lyytimäki, 2013; Lewanzik & Voigt, 2014). Light pollution has increased considerably and continues to increase worldwide, often associated with urban development (Cinzano *et al.,* 2001; Bruce-White & Shardlow, 2011), although levels may be declining in some economically developed regions (Bennie *et al.,* 2014). The predominant types of artificial lighting in use are also changing; lights emitting a broader spectrum of wavelengths are increasingly favoured because they facilitate human discernment of colours at night and, in the case of light-emitting diodes (LEDs), are more energy-efficient (Bruce-White & Shardlow, 2011; Gaston *et al.,* 2012).

Artificial night lighting, even at low levels, exerts an influence at every level of biological organisation (Gaston *et al.,* 2013), from cell (Navara & Nelson, 2007) to organism (Longcore & Rich, 2004) and community (Davies *et al.,* 2012). However, little is currently known about the effects of light pollution on species population dynamics, whole communities, and networks of interacting species, or ecosystem functioning.

Long-term declines in populations and distributions of many moth species have been found in Great Britain (Conrad *et al.,* 2004, 2006; Fox *et al.,* 2011, 2013), the Netherlands (Groenendijk & Ellis, 2011), and Finland (Mattila *et al.,* 2006, 2008). Habitat degradation and climate change are likely drivers of these declines (Fox *et al.,* 2014), as with diurnal pollinators (Potts *et al.,* 2010); however, artificial night lighting has also been proposed as a potential contributing factor (Fox, 2013; Fox *et al.,* 2013). Conrad *et al*. (2006) found no significant correlation between a change in light pollution and a change in light-trap catches from 1992 and 2000, but short-term trends in moth (and other insect) populations can be difficult to detect, as large inter-annual fluctuations are normal (Conrad *et al.,* 2004).

Below, we describe a range of mechanisms by which artificial night lighting could impact negatively upon moths. Many such impacts are not empirically proven. Therefore, we describe first the well-established mechanisms, followed by those unproven, but for which some evidence exists. Even where negative impacts have been demonstrated, their effects at the population level are mostly unknown.

## Established effects of artificial light on moths

Individual moths are certainly affected by artificial night lighting, famously appearing to be attracted to artificial lights, sometimes in huge numbers (Howe, 1959). Numerous theories have been put forward to explain flight-to-light behaviour (Robinson & Robinson, 1950; Mazokhin-Porshnyakov, 1961; Callahan, 1965; Hsiao, 1973; Sotthibandhu & Baker, 1979; Hamdorf & Höglund, 1981), although the debate is inconclusive. Nevertheless, this observation has led to the popularity of using light-baited traps to survey many families of moths.

The extent to which moths are attracted to light varies according to a number of factors. It has been recognised for many years that shorter wavelengths are, in general, more attractive to moths (Frank, 2006, and references therein); attractiveness appears to peak around wavelengths of 400 nm (violet light; Cowan & Gries, 2009). The degree of attraction and preferred wavelengths both vary between moth taxa (Merckx & Slade, 2014); typically, larger-bodied moths with larger eyes are more likely to be attracted to light dominated by smaller wavelengths (van Langevelde *et al.,* 2011; Somers-Yeates *et al.,* 2013). Variation also appears to exist between sexes; males of some species are significantly more likely to be recorded at light traps than females (Garris & Snyder, 2010), but it is not clear if this is due to stronger male attraction to lights, or males being more active and therefore more likely to move into the zone of influence of a given light (Altermatt *et al.,* 2009).

Aside from flight-to-light behaviour, moths may be further affected by artificial night lighting through other mechanisms, related to direct interaction with lights, increased ambient light at night, and locally altered perception of photoperiods in the vicinity of artificial lights. Contact with hot components of lamps or radiant energy from bright lights can kill insects or damage their wings, legs, and antennae (Eisenbeis, 2006; Frank, 2006). Insects killed by light-baited electric traps, primarily targeting biting Diptera, contain a high proportion of nocturnal Lepidoptera (Frick & Tallamy, 1996).

### Reproduction

Reproductive success of moths could also be negatively affected by artificial night lighting. Low levels of artificial light inhibited the release of sex pheromones by female moths of a Geometridae species (Sower *et al.,* 1970). Artificial light can suppress oviposition (Nemec, 1969) or act as an ecological trap, causing females to lay eggs at an unusually high density and/or in unsuitable locations near to lights (Pfrimmer *et al.,* 1955; Brown, 1984), either of which could increase larval competition for limited food resources.

Artificial light may also have an effect on larvae, which are nocturnal in many Lepidopteran species, including some that are diurnal as adults (butterflies and day-flying moths). Even at a low intensity, light caused reductions in age and mass at pupation in males and inhibited diapause in both sexes of a Noctuidae species in the laboratory (van Geffen *et al.,* 2014). However, few studies have investigated the effects of artificial night lighting on Lepidopteran larvae.

### Predation

Predators of moths have been observed to hunt at artificial lights, exploiting above-average prey densities caused by flight-to-light behaviour (Frank, 2006). This includes both active hunters, such as bats (Rydell, 1992) and predatory insects (Warren, 1990), and sit-and-wait predators, such as spiders (Heiling, 1999), reptiles, and amphibians (Henderson & Powell, 2001). Artificial light also interferes with the anti-bat defensive behaviour of moths, increasing their vulnerability to predation (Svensson & Rydell, 1998; Acharya & Fenton, 1999).

## Possible further effects of artificial light on moths

In addition to the known mechanisms described above, a number of other mechanisms have the potential to affect moths but have not yet been conclusively demonstrated.

### Reproduction

Changes in photoperiod disrupted the pheromone release behaviour of females of a Pyralidae species (Fatzinger, 1973), which could disrupt mating. Competition in male moths between light traps and pheromone traps (Delisle *et al.,* 1998) suggests that artificial lighting could distract males from female pheromone signals and thus reduce mating frequency. More severely, radiant energy from bright lights can sterilise other insects in the laboratory (Riordan, 1964; Eisenbeis, 2006); this could occur with moths in the wild. Artificial lights have been observed to divert dispersing or migrating moths to locations that are unsuitable for breeding (Frank, 2006, and references therein), potentially creating an ecological trap.

A reduction of the dark scotophase of the photoperiod prevented diapause in the larval stage of a Tortricidae species in the laboratory (Berlinger & Ankersmit, 1976); however, this result could not be replicated in field trials. In addition, moth larvae may be attracted to artificial lights in much the same way as adults (Gillett & Gardner, 2009).

### Predation

Artificial light may also increase the risk of predation by disrupting crypsis, both by causing moths to rest in unsuitable locations where their wing patterns are an ineffective disguise, and by concentrating moths in a small area, assisting predators in establishing a search image of cryptic wing patterns (Frank, 2006). Similarly, repeat exposure can habituate predators to stimuli that elicit startle reactions, such as patterned hindwings or bodies (Schlenoff, 1985; Ingalls, 1993); highly visible aggregations of moths around lights could accelerate the habituation process (Frank, 2006).

### Vision

Artificial light affects the sensitivity of the compound eyes of moths (Frank, 2006). Screening pigment reduces ocular sensitivity within 23 min of exposure to light (Hamdorf & Höglund, 1981); the return to full ocular sensitivity is far slower, taking around 30 min (Bernhard & Ottoson, 1960). To what extent these effects may be exerted by exposure to artificial lights in natural settings is unclear. However, moths attracted to a light will often rest on vegetation or the ground for a period of time, sometimes before even reaching the light (Hartstack *et al.,* 1968; Hsiao, 1973); this behaviour could represent a period of readjustment to full ocular sensitivity.

In addition to compound eyes, most insects (including moths) have simple eyes (dorsal ocelli) that are sensitive to changes in light intensity (Mizunami, 1995), and appear to have a role in timing flight initiation at dusk in moths (Eaton *et al.,* 1983). It is possible that artificial night lighting could delay or even prevent the onset of nocturnal activity. While this effect is likely to be localised to the immediate vicinity of light sources, it could negatively affect moth fitness (and hence population growth) and nocturnal pollination.

The visual capacity of moths could also be indirectly affected by artificial night lighting altering the spectrum of background illumination. Ultraviolet (UV) radiation (10–400 nm), predominantly at longer wavelengths close to visible light (Eguchi *et al.,* 1982), is particularly important to pollinating moths, as moths orient themselves to flowers by a combination of olfactory and visual cues (Raguso & Willis, 2005) including UV-reflecting markers on flowers (Barth, 1985). The spectral content of artificial night lighting will therefore determine its effect upon flower-visiting moths (Davies *et al.,* 2013): UV-rich lighting (e.g. from mercury vapour lights) could accentuate these nectar guides, whereas UV-poor lighting (e.g. from low-pressure sodium lights), by illuminating other parts of the nocturnal environment relatively more brightly, could cause nectar guides to stand out less clearly (Frank, 2006).

## Moths and pollination: an ecological network approach

The studies above considered the direct effects of artificial light upon moths, mostly at the level of the individual. Whether artificial night lighting, through these effects, is a contributing factor in declines in moth populations remains a key research question. It is also necessary to consider the indirect effects of artificial light mediated by moth pollination, as can be demonstrated with an ecological network approach. Ecological networks describe the structure of communities as the occurrence (and frequency) of interactions between species, such as plants and pollinators (Montoya *et al.,* 2006; Bascompte, 2007). From descriptions of the network's structure, its function can be inferred (Tylianakis *et al.,* 2010); for example, its robustness to perturbations such as species extinction and their cascading effects (Bascompte, 2009; Ings *et al.,* 2009; Evans *et al.,* 2013). It has been demonstrated that drivers of environmental change, such as climate change, can alter the composition and balance of networks (Tylianakis *et al.,* 2008), including plant–pollinator networks (Rathke & Jules, 1993; Memmott *et al.,* 2007). Removal of pollinator species can cause plant species diversity to suffer (Memmott *et al.,* 2004; Fontaine *et al.,* 2006), while loss of plants can likewise affect pollinators (Wallis De Vries *et al.,* 2012).

Two attributes of networks are particularly important. First, many pollinator networks have a nested structure, in which specialist species (with few connections in the network) tend to interact with generalists (with many connections) more frequently than with other specialists (Dicks *et al.,* 2002; Bascompte *et al.,* 2003). Nested systems have high tolerance to the random loss of species from the community but are sensitive to the removal of certain highly connected species (Solé & Montoya, 2001; Memmott *et al.,* 2004). Second, these systems are also modular, in which sets of species within modules interact strongly with each other; these modules are akin to pollination syndromes (Olesen *et al.,* 2007), and increase overall robustness because impacts cascade less quickly between modules and through the whole system. Some modules are as a result of close co-evolutionary relationships; in extreme examples, plants are entirely reliant on a single or few species of moth [eg. *Oxyanthus pyriformis* (Hochst.) Skeels (Johnson *et al.,* 2004)]. In such cases, minor disruption of the pollinator will directly impact the reproductive success of the plant (Pauw, 2007). The modules themselves may be nested within the whole system, and species will often be nested within modules.

Most studies of plant–pollinator networks to date have focused on diurnal interactions. Two exceptions considering nocturnal plant–pollinator networks are Devoto *et al*. (2011) and Banza (2011); these authors identified nocturnal moth–flower interactions by sampling pollen on captured moths. Combining nocturnal pollination networks with diurnal ones could lead to increased modularity (if there are distinct sets of flowers visited by diurnal and nocturnal pollinators), such that the effects of environmental change (e.g. artificial night lighting) could be substantial in one part of the network but not cascade through the whole network. It could also lead to increased redundancy (if flowers share diurnal and nocturnal pollinators), such that the plants in the network may be robust to the disruption of one set of pollinators (e.g. moths). Testing for differences in the structure of plant–moth pollinator networks between unlit and artificially lit sites will begin to empirically reveal the functional impact of artificial night lighting on wider communities through indirect, as well as direct, effects.

## Potential effects of artificial light on moth pollination

**Fig 2:**
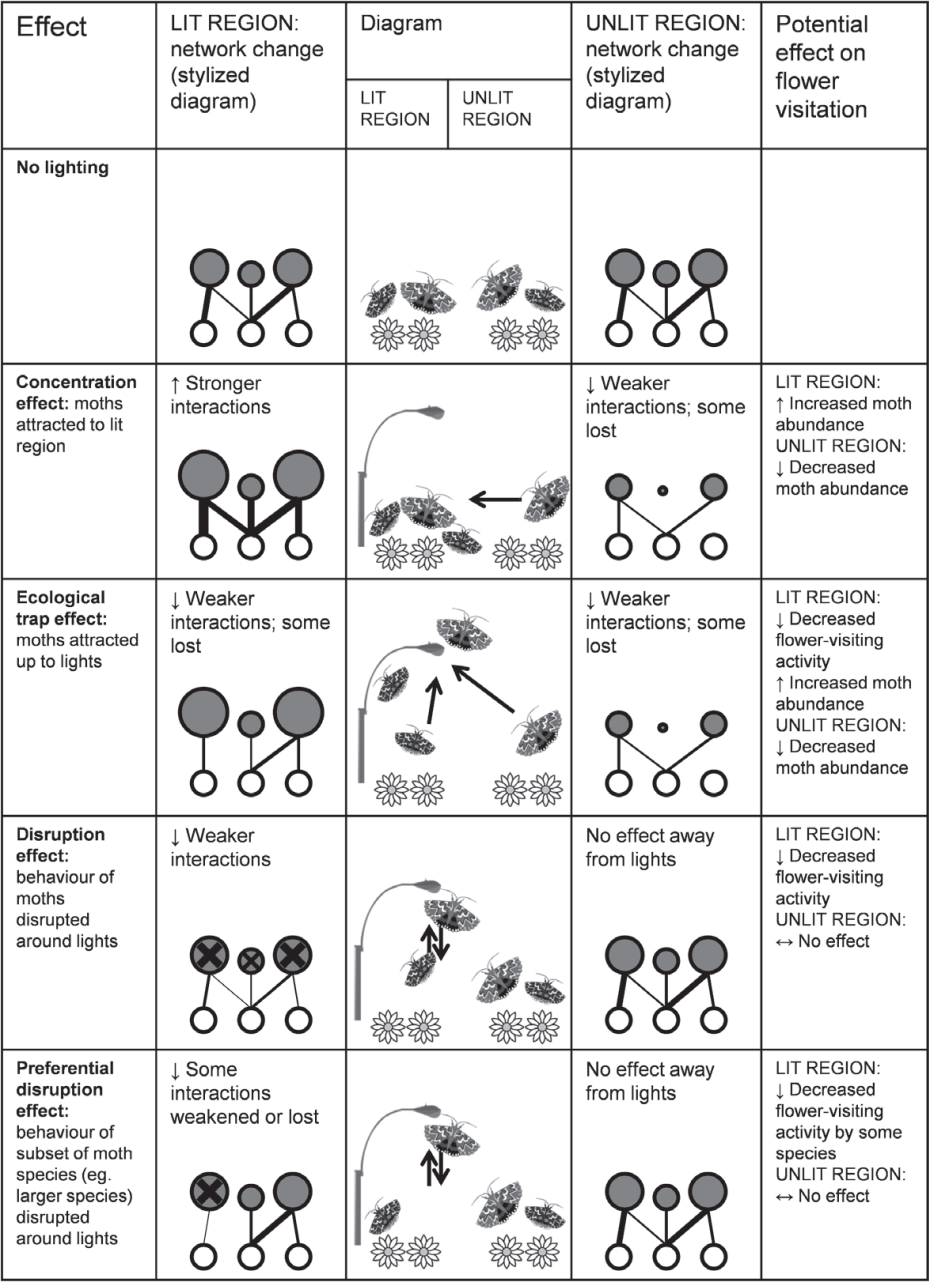
Possible scenarios for change in plant–moth pollination networks as a result of artificial night lighting, with predictions for effects on local flower-visitation activity by moths. In network representations, nodes represent species (lower = flowering plants, upper = moths) and links represent pollination interactions. Node width represents relative species abundance and link thickness represents interaction strength. Crosses indicate disruption of behaviour.

A variety of changes in moth abundance, composition of moth assemblages, and moth behaviour are all possible results of artificial lighting at night, but the overall effect on the whole community via disruption of pollination remains to be tested (Fig. 2). Moths may be drawn in towards a light from several metres away (Baker & Sadovy, 1978; Truxa & Fiedler, 2012; van Grunsven *et al.,* 2014); this might alter local moth abundance and the composition of moth assemblages both in the vicinity of lights, and in the source habitats from which attracted moths are drawn (Fig. 2: concentration and ecological trap effects). Interactions could also be weakened or lost through behavioural changes in moths, even if their abundance is unchanged (Fig. 2: disruption effect). The level and nature of disruption might vary between moth species (van Langevelde *et al.,* 2011; Somers-Yeates *et al.,* 2013), leading to some interactions being more strongly affected than others (Fig. 2: preferential disruption effect). If reproduction is affected, some moth species may decline in abundance or go extinct, leading to further loss of interactions. Therefore, the effects of increasing artificial light may be positive for some moth or plant species and negative for others in any given community, leading to cascading changes in the system that are difficult to predict prior to empirical, experimental research.

## Discussion

### Future research directions

**Fig 3:**
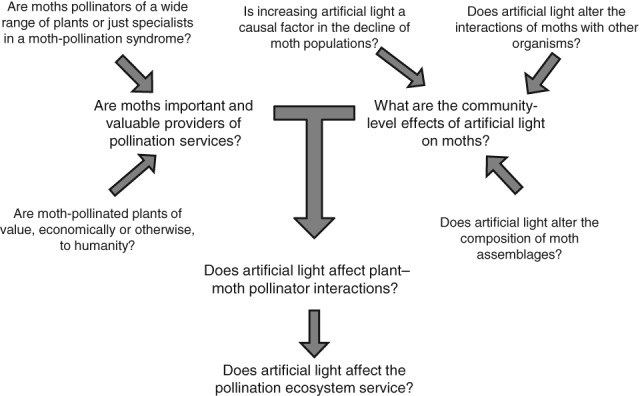
Future research directions raised in this review.

We believe that our findings in this review highlight a number of key priorities for future research (Fig. 3). While we have described evidence that moths are pollinators of a diverse range of plant species, the extent of their role as pollinators in maintaining botanical diversity, in agro-ecosystems, and especially of commercially valuable crops demands attention.

The effects of artificial night lighting on moths, too, should be investigated further. Many of the individual-level effects summarised above have not been empirically demonstrated to occur under natural conditions. Moreover, there are no published studies into the community-level effects of artificial night lighting on moths; this is a major research gap (Fox, 2013; Gaston *et al.,* 2013). The impacts of lighting on plant–moth pollination networks are difficult to predict (Fig. 2) and also require empirical testing. It is worth noting that moths are a food source for many other organisms including birds and bats (Fox, 2013); therefore, a similar approach with trophic networks may also be worthwhile.

### Conclusion

In this review, we show the importance of moths as pollinators for a diverse range of plant species in ecosystems worldwide and, hence, their role in ecosystem functioning. We discuss the many ways in which moths are known to be affected by artificial night lighting, and suggest how these effects may, in turn, impact pollination interactions between moths and plants.

The effects of artificial night lighting may go beyond simple declines in moth populations, with potential changes in the composition of moth assemblages and in the nature and frequency of interspecies interactions between moths and other taxa; this justifies an ecological network approach to the problem (Fig. 2).

Artificial night lighting may negatively affect a range of ecosystem services (Lyytimäki, 2013; Lewanzik & Voigt, 2014). Based on the evidence summarised in this review, we consider pollination to be one such ecosystem service that may be disrupted by increasing ecological light pollution. The research directions outlined will help develop an understanding of what form that disruption may take, and may direct ways to mitigate the negative effects of artificial night lighting upon moths and the ecosystem processes that rely upon them.

## Supporting Information

**Appendix S1.** References for Fig. 1.

**Appendix S2.** Further tables summarising results of the moth-pollination review.
