# Supplementary material for: Pollination by nocturnal Lepidoptera, and the effects of light pollution: a review
Source: Ecol Entomol. 2014 Dec 13;40(3):187–98. doi: 10.1111/een.12174 (PMC4405039; doi:10.1111/een.12174)
Supplement: Supplementary file 1 — Appendix S1. References for Fig. 1. [file een0040-0187-sd1.docx]

**Appendix S1: References for Figure 1.**

Drawings are used under a ClipArt ETC Paid Commercial License (<http://etc.usf.edu/clipart>). The source of each image, numbered clockwise from the hoverfly, is:

1: Whitney, W.D., ed. (1889)*The Century Dictionary and Cyclopedia.* New York, NY: The Century Co.

2, 6: Smith, J.B. (1896) *Economic Entomology*. Philadelphia, PA and London: J.B. Lippincott Co.

3, 5: Cuppy, H.A., ed. (1895) *Beauties and Wonders of Land and Sea.* Springfield, OH: Mast, Crowell & Kirkpatrick.

4, 13: Nicholson, G. (1884) *The Illustrated Dictionary of Gardening, Div. VI.* London: L. Upcott Gill.

7: *The Encyclopedia Britannica, New Werner Edition* (1893). New York, NY: The Werner Company.

8: Lindley, J. (1853) *The Vegetable Kingdom.* London: Bradbury & Evans.

9: Foster, E.D., ed. (1921) *The American Educator (vol. 1).* Chicago, IL: Ralph Durham Company.

10, 12, 15: Mathews , F.S. (1902) *Field Book of American Wild Flowers.* New York, NY: G. P. Putnam's Sons.

11: Bailey, L.H. (1917) *Standard Cyclopedia of Horticulture.* New York, NY: The MacMillan Company.

14: No source provided.

16: *The Encyclopedia Britannica, Eleventh Edition* (1910) New York, NY: The Encyclopedia Britannica Company.
