# Supplementary material for: Pollination by nocturnal Lepidoptera, and the effects of light pollution: a review
Source: Ecol Entomol. 2014 Dec 13;40(3):187–98. doi: 10.1111/een.12174 (PMC4405039; doi:10.1111/een.12174)
Supplement: Supplementary file 2 — Appendix S2. Further tables summarising results of the moth-pollination review. [file een0040-0187-sd2.docx]

**Appendix S2**

**Table S1:** Summary of reviewed studies of the role of moths in pollination (see Tables S1.1–1.3).

| Type of moth-pollination study | No. studies | No. ecosystems, species, or taxa |
| --- | --- | --- |
| Ecosystems (Table S1.1) | 14 | 13 |
| Plant species (Table S1.2) | 143 | 289 |
| Pollinating seed parasites (Table S1.3) | 11 | 12 |
| Total | 168 | 314 |

**Table S1.1:** Ecosystems in which moths have been found to be important pollinators.

In column 5 (Methods), the methods used to provide evidence for moth pollination are indicated as follows: C = contact with anthers and/or stigmas observed, D = pollen deposited on and/or removed from stigmas, E = experimental exclusion of diurnal and nocturnal pollinators, I = inferred by pollination syndrome, P = pollen present on captured moths, R = literature review, S = moth scales or hairs present on stigmas, VF = flower visitation determined by fluorescent markers deposited by visiting moths, VO = flower visitation determined by observations, VR = flower visitation determined by video recordings U = unspecified/unavailable.

| Climate | System | Location | % of plants moth-pollinated | Methods | Notes | Reference |
| --- | --- | --- | --- | --- | --- | --- |
| Temperate | Coniferous forest | Scotland |  | P | ~25% of moths were carrying pollen. | Devoto *et al.* (2011) |
|  | Various | South-eastern Africa |  | R | Moth pollination is relatively common in some areas as bee diversity is low. | Johnson (2004) |
|  | Meadow | Portugal |  | P | ~39% of moths were carrying pollen representing 36 plant taxa. | Banza (2011) |
|  | Xeric sandhill | Florida, USA |  | P, VO, VR |  | Atwater (2013) |
| Tropical | Bush-savannah | Kenya | ~4.6 | I, VO | Sphingidae only investigated. | Martins and Johnson (2013) |
|  | Grassland | Venezuela | 6 | C, P, VO | Moth-pollinated plants the second most abundant animal-pollinated group. | Ramirez (2004) |
|  | Lowland dry forest | Costa Rica | ~10 | P, VO | Sphingidae only investigated. | Haber and Frankie (1989) |
|  | Monsoon forest | Laos | 6 | P, VO |  | Kato *et al.* (2008) |
|  | Oceanic islands | Galápagos |  | R | Only Hymenoptera more important to pollination than moths. | Chamorro *et al.* (2012) |
|  | Rainforest | Costa Rica | 16 | I, VO | Moth-pollinated plants the second most abundant group. | Bawa *et al.* (1985) |
|  | Rainforest | New Caledonia | 20 | P, VO | Moth-pollinated plants the second most abundant group. | Kato and Kawakita (2004) |
|  | Savannah | Brazil | 14 | I, VO | Woody plants only; moths were third most important pollinators. | Oliveira *et al.* (2004), Martins and Batalha (2006) |
|  | Wet evergreen forest | India |  | C, VO | Moths are third most important pollinators. | Devy and Davidar (2003) |

**Table S1.2:** Examples of plants found to be pollinated by moths.

In column 3 (Prediction of moth pollination): E = explicit (stated) prediction of pollination by hawkmoths (Sphingidae), I = implicit prediction of pollination by hawkmoths (eg. introductory discussion of characteristics of sphingophilous flowers), N = prediction of no pollination by moths, O = prediction of pollination by moths other than hawkmoths, U = prediction of pollination by general or unspecified moths, and X = no clear prediction made.

In column 4 (Moth pollinators): C = Cosmopterigidae, Cr = Crambidae, Ct = Ctenuchidae, E = Erebidae, Ge = Gelechiidae, G = Geometridae, Gl = Glyphipterigidae, Gr = Gracillariidae, L = Lasiocampidae, M = Micropterigidae, N = Noctuidae, No = Nolidae, Pr = Prodoxidae, Pt = Pterophoridae, P = Pyralidae, Sa = Saturniidae, Se = Sesiidae, S = Sphingidae, Th = Thyrididae, T = Tortricidae, U = Uranidae, X = unspecified/unknown. For studies where exact pollinating moth species or genera are given, this is detailed in column 7 (Notes).

In column 6 (Methods), the methods used to provide evidence for moth pollination are indicated as follows: C = contact with anthers and/or stigmas observed, D = pollen deposited on stigmas and/or removed from anthers, E = plants pollinated when experimentally exposed only to visits by moths, I = inferred by pollination syndrome, P = pollen present on captured moths, S = moth scales or hairs present on stigmas, VF = flower visitation determined by fluorescent markers transferred by visiting moths, VO = flower visitation determined by observations, VR = flower visitation determined by video recordings, VT = flower visitation determined by flower-visitor trapping, U = unspecified/unavailable.

| Plant Family | Plant species | Prediction of moth pollination | Moth pollinators | Other pollinators? | Methods | Notes | References |
| --- | --- | --- | --- | --- | --- | --- | --- |
| Adoxaceae | *Adoxa moschatellina* L. | N | N | Various Diptera | P | *Orthosia gothica* L. was the main nocturnal pollinator. Three other *Orthosia* spp. and *Lithophane hepatica* Clerck. also recorded carrying pollen. | Holmes (2005) |
| Amaranthaceae | *Beta vulgaris* L. | U | X | – | P, VO |  | Banza (2011) |
| Amaryllidaceae | *Allium cepa* L. | U | X | – | P, VO |  | Banza (2011) |
|  | *Ammocharis tinneana* (Kotschy & Peyr.) Milne-Redh. & Schweick. | E | S | – | VO |  | Martins and Johnson (2013) |
|  | *Crinum flaccidum* Herb. | E | S | None | I |  | Howell and Prakash (1990) |
|  | *Crinum jagus* (J. Thomps.) Dandy | E | S | None | C, VO | Only Sphingidae considered as potential pollinators | Brantjes and Bos (1980) |
|  | *Crinum macowanii* Baker | E | S | – | VO |  | Martins and Johnson (2013) |
|  | *Hymenocallis coronaria* (Leconte) Kunth | E | N, S | Hymenoptera, Trochilidae | C, VO |  | Graham (2010) |
|  | *Hymenocallis occidentalis* (Leconte) Kunth | E | E, N, S | None | C, VO |  | Graham (2010) |
|  | *Narcissus papyraceus* Ker Gawl. | E | S, X | Syrphid flies (Diptera) | P |  | Pérez-Barrales *et al.* (2007) |
|  | *Narcissus viridiflorus* Schousb. | O | X | None | I |  | Vogel and Mueller-Doblies (1975) |
|  | *Pancratium maritimum* L. | I | S | None | P, VF, VO |  | Eisikowitch and Galil (1971) |
| Anacardiaceae | *Schinus terebinthifolia* Raddi | X | X | Hymenoptera | VO |  | Kato and Kawakita (2004) |
| Apiaceae | *Daucus carota* L. | U | X | *–* | P, VO |  | Banza (2011) |
| Apocynaceae | *Acokanthera schimperi* (A.DC.) Schweinf. | E | S | – | VO |  | Martins and Johnson (2013) |
|  | *Alstonia costata* (G.Forst.) R.Br. | X | X | None | VO |  | Kato and Kawakita (2004) |
|  | *Asclepias syriaca* L. | U | E, G, N | *Bombus* spp. (Hymenoptera: Apidae) | P, VO |  | Jennersten and Morse (1991) |
|  | *Asclepias verticillata* L. | U | G, N | *Bombus* spp. | P, VO |  | Bertin and Willson (1980) |
|  | *Aspidosperma macrocarpon* Mart. | U | X | None | I, VO | Not Sphingidae | Oliveira *et al.* (2004), Martins and Batalha (2006) |
|  | *Aspidosperma nobile* Müll.Arg. | U | X | – | I |  | Martins and Batalha (2006) |
|  | *Aspidosperma polyneuron* Müll.Arg. | U | X | – | I |  | Martins and Batalha (2006) |
|  | *Aspidosperma quebracho-blanco* Schltdl. | U | N, P | None | C, VO |  | Lin and Bernardello (1999) |
|  | *Aspidosperma tomentosum* Mart. | U | X | None | I, VO | Not Sphingidae | Oliveira *et al.* (2004), Martins and Batalha (2006) |
|  | *Carissa spinarum* L. | E | S | – | VO |  | Martins and Johnson (2013) |
|  | *Cerbera manghas* L. | X | S | None | VO |  | Kato and Kawakita (2004) |
|  | *Chonemorpha fragrans* (Moon) Alston | X | S | None | VO |  | Kato *et al.* (2008) |
|  | *Hancornia speciosa* Gomes | U | S | None | I, VO |  | Oliveira *et al.* (2004), Martins and Batalha (2006) |
|  | *Himatanthus obovatus* (Müll.Arg.) Woodson | U | S | None | I, VO |  | Oliveira *et al.* (2004), Martins and Batalha (2006) |
|  | *Mandevilla laxa* (Ruiz & Pav.) Woodson | I | S | None | P | Primarily *Manduca sexta* L. | Moré *et al.* (2007) |
|  | *Mandevilla longiflora* (Desf.) Pichon | I | S | None | P | Primarily *Manduca sexta* | Moré *et al.* (2007) |
|  | *Mandevilla petraea* (A. St.-Hil.) Pichon | I | S | None | P | Primarily *Manduca tucumana* Rothschild & Jordan | Moré *et al.* (2007) |
|  | *Metaplexis japonica* (Thunb.) Makino | U | N, P | None | P, VO |  | Sugiura and Yamazaki (2005) |
|  | *Sarcostemma angustissimum* (Andersson) R.W. Holm | N | N, T | None | VO |  | Philipp *et al.* (2006) |
|  | *Strophanthus wallichii* A.DC. | X | X | None | VO |  | Kato *et al.* (2008) |
| Arecaceae | *Elaeis guineensis* Jacq. | N | C | Thysanoptera | VO |  | Syed (1979) |
| Asparagaceae | *Agave lechuguilla* Torr. | N | S | Various Hymenoptera | C, VO | Primarily *Hyles lineata* Fabricius | Silva-Montellano and Eguiarte (2003) |
|  | *Agave macroacantha* Zucc. | N | N, S, X | Bats (Chiroptera), Hymenoptera, diurnal Lepidoptera, hummingbirds (Trochilidae) | VO | Extremely dependent on nocturnal pollinators (probably Chiroptera) for reproductive success | Arizaga *et al.* (2000a, 2000b) |
|  | *Agave palmeri*  Engelm. | N | S | Bats (Chiroptera) | P |  | Alarcón *et al.* (2008) |
|  | *Chlorogalum pomeridianum* (DC.) Kunth | E | S | – | P, VO |  | Grant (1983) |
|  | *Manfreda virginica* L. Salisb. ex Rose | I | N, S | Large bees (Hymenoptera) | C, VO | Behavioural observations indicate Noctuidae unlikely to contribute significantly to pollination | Groman and Pellmyr (1999) |
|  | *Ornithogalum narbonense* L. | U | X | – | P, VO |  | Banza (2011) |
|  | *Yucca* spp. | X | Pr | – | U | *Tegeticula* and *Parategeticula* spp. Obligate pollinating seed parasite mutualism | Pellmyr *et al.* (1996) |
| Asteraceae | *Ageratina aromatica* (L.) Spach | U | X | *–* | P, VO |  | Atwater (2013) |
|  | *Balduina angustifolia* (Pursh) B.L.Rob. | U | X | *–* | P, VO |  | Atwater (2013) |
|  | *Cirsium* spp. | U | N | *–* | P | *Diarsia mendica mendica* Fabricius. Pollen of *Cirsium palustre* (L.) Coss. ex Scop. and *C. arvense* (L.) Scop. not distinguished | Devoto *et al.* (2011) |
|  | *Espeletia grandiflora* Humb. & Bonpl. | N | G, N, P | *Bombus* spp. (Apidae) primarily, as well as Trochilidae, Diptera and Coleoptera | C, VO |  | Fagua and Gonzalez (2007) |
|  | *Eupatorium compositifolium* Walter | U | X | *–* | P, VO |  | Atwater (2013) |
|  | *Galactites tomentosa* Moench. | U | X | *–* | P, VO |  | Banza (2011) |
|  | *Glebionis coronaria* (L.) Cass. ex Spach | U | X | *–* | P, VO |  | Banza (2011) |
|  | *Jacobaea vulgaris* Gaertn. | U | G, N | *–* | P |  | Devoto *et al.* (2011) |
|  | *Leontodon taraxacoides* Hoppe & Hornsch. | U | X | *–* | P, VO |  | Banza (2011) |
|  | *Liatris tenuifolia* Nutt. | U | X | *–* | P, VO |  | Atwater (2013) |
|  | *Pityopsis graminifolia* (Michx.) Nutt. | U | X | *–* | P, VO |  | Atwater (2013) |
|  | *Senecio vulgaris* L. | U | X | *–* | P, VO |  | Banza (2011) |
|  | *Tithonia diversifolia* (Hemsl.) A.Gray | E | S | – | VO |  | Martins and Johnson (2013) |
| Balsaminaceae | *Impatiens coelotropis* Fischer | X | S | Diurnal Lepidoptera, Hymenoptera and Diptera | C, P, VO |  | Sreekala *et al.* (2008) |
|  | *Impatiens cuspidata* Wight & Arn. | X | S | Diurnal Lepidoptera, Hymenoptera and Diptera | C, P, VO |  | Sreekala *et al.* (2011) |
| Bignoniaceae | *Catalpa speciosa* (Warder ex Barney) Warder ex Engelm. | U | E, G, L, N | Hymenoptera | S, VO |  | Stephenson and Thomas (1977) |
|  | *Pyrostegia millingtonioides* Sandwith | U | X | None | I | Flowers fit moth-pollination syndrome and are most likely moth-pollinated | Pool (2008) |
|  | *Sphingiphila tetramera* A. Gentry | X | S | None | I |  | Gentry (1990) |
| Boraginaceae | *Cordia revoluta* Hook.f. | N | P, X | None | VO |  | Philipp *et al.* (2006) |
|  | *Cynoglossum creticum* Mill. | U | X | *–* | P, VO |  | Banza (2011) |
|  | *Macromeria viridiflora* A. DC. | E | S | Trochilidae | D, VO | Primarily by Trochilidae | Boyd (2004) |
|  | *Tournefortia rufo-sericea* Hook. f. | U | N, P | Ants (Hymenoptera) and Coleoptera | P, VO | Ants are primary pollinators | McMullen (2007) |
| Brassicaceae | *Maerua decumbens* (Brongn.) DeWolf | E | S | – | VO |  | Martins and Johnson (2013) |
|  | *Raphanus raphanistrum* L. | U | X | *–* | P, VO |  | Banza (2011) |
|  | *Rapistrum rugosum* (L.) All. | U | X | *–* | P, VO |  | Banza (2011) |
| Cactaceae | *Cereus repandus* (L.) Mill. | X | S | None | I, VO | *Agrius cingulata* Fabricius and *Manduca rustica* Fabricius | Silva and Sazima (1995) |
|  | *Echinopsis ancistrophora* Speg. | E | S | Solitary bees (Hymenoptera) | P, S | Populations with long flower tubes moth-pollinated; populations with short flower tubes bee-pollinated | Schlumpberger *et al.* (2009) |
|  | *Echinopsis schickendantzii* F.A.C. Weber | U | S | Hymenoptera | P, S, VO |  | Alonso-Pedano and Ortega-Baes (2012) |
|  | *Echinopsis terscheckii* (Parm.) Friedrich & G.D. Rowley | U | G, N, Sa, S | Hymenoptera and Aves | P, S | Moths are the most effective pollinators | Ortega-Baes *et al.* (2011) |
|  | *Lophocereus schottii* (Engelm.) Britton & Rose | X | P | – | E, VO | *Upiga virescens* Hulst. Obligate pollinating seed parasite mutualism | Fleming and Holland (1998) |
|  | *Peniocereus striatus* (Brandegee) Buxb. | I | S | None | VF, VO | *Hyles lineata* and *Manduca quinquemaculata* Haworth | Suzán *et al.* (1994) |
|  | *Selenicereus wittii* (K. Schum.) G.D. Rowley | X | S | None | I | Pollination by either or both of *Cocytius cluentus* Cramer and *Amphimoea walkeri* Boisduval, the only species in the plant’s range with sufficiently long proboscides | Barthlott *et al.* (1997) |
| Capparaceae | *Crateva religiosa* G. Forst. | X | P | Hymenoptera | P, VO | *Achoria grisella* Fabricius | Sharma *et al.* (2006) |
| Caprifoliaceae | *Fedia cornucopiae* (L.) Gaertn. | U | X |  | P, VO |  | Banza (2011) |
|  | *Lonicera japonica* Thunb. | I | N, S | *Lasioglossum* spp. (Hymenoptera: Halictidae) | D, VO | Primarily *Theretra japonica* Boisduval (Sphingidae) | Miyake and Yahara (1998) |
|  | *Valerianella discoidea* (L.) Loisel. | U | X | *–* | P, VO |  | Banza (2011) |
| Caricaceae | *Jacaratia spinosa* (Aubl.) A. DC. | I | X | Diurnal Lepidoptera | I, VO | Moths are primary pollinators | Piratelli *et al.* (1998) |
| Caryocaraceae | *Caryocar brasiliense* A. St.-Hil. | I | S | Chiroptera | P, VO | Chiroptera are major pollinators | Gribel and Hay (1993) |
| Caryophyllaceae | *Dianthus gratianopolitanus* Vill. | U | N, S | Diurnal Lepidoptera | VO |  | Erhardt (1990) |
|  | *Dianthus superbus* L. | I | N, S | None | VO |  | Erhardt (1991) |
|  | *Dianthus sylvestris* Wulfen | O | N, S | Hymenoptera, Diptera | VO | *Hadena compta* Denis & Schiffermüller (Noctuidae), *Macroglossum stellatarum* L. (Sphingidae). Pollinating seed parasite mutualism with *H. compta* | Collin *et al.* (2002) |
|  | *Saponaria officinalis* L. | I | N, S | None | VO |  | Wolff *et al.* (2006) |
|  | *Schiedea lydgatei* Hillebr. | N | P | Wind | P, VO |  | Norman *et al.* (1997) |
|  | *Silene dioica* (L.) Clairv. | O | G | – | P, VO | *Perizoma affinitatum* Stephens. Pollinating seed parasite mutualism | Westerbergh (2004) |
|  | *Silene latifolia* Poir. | I | N, S | Thysanoptera | U | Generally pollinated by moths, which are the best pollinators of this species | McNeill (1977), Young (2002) |
|  | *Silene sennenii* Pau | U | Cr, G, N, S | Various Hymenoptera and Diptera | VO | Pollination shown to occur mainly at night | Martinell *et al.* (2010) |
|  | *Silene stellata* (L.) W.T. Aiton | O | N | – | D | *Hadena ectypa* Morrison. Pollinating seed parasite mutualism | Kula *et al.* (2013) |
|  | *Silene succulenta* Forssk. | I | S | None | P, VF | Not the focal species of this study | Eisikowitch and Galil (1971) |
|  | *Silene viscaria* (L.) Jess. | N | G, N, S | Various Hymenoptera and Diptera | VO | Primarily *Deilephila porcellus* L. (Sphingidae) | Jennersten (1988) |
|  | *Silene vulgaris* (Moench) Garcke | U | N, S | Various Hymenoptera and Diptera | P, VO | 24 spp. Noctuidae and 2 spp. Sphingidae | Pettersson (1991) |
| Cleomaceae | *Cleome gynandra* L. | E | S | – | VO |  | Martins and Johnson (2013) |
| Convulvulaceae | *Convolvulus althaeoides* L. | U | X | *–* | P, VO |  | Banza (2011) |
|  | *Ipomoea ampullacea* Fernald | X | X | None | I |  | Wilkin (1995) |
|  | *Ipomoea habeliana* Oliv. | I | S | None | P, VF, VO | Though other taxa are flower visitors, only Sphingidae are effective pollinators | McMullen (2009) |
|  | *Merremia palmeri* (Hallier) Hallier f. | E | S | None | D, VO |  | Willmott and Burquez (1996) |
| Crassulaceae | *Crassula fascicularis* Lam. | X | G | None | P |  | Johnson *et al.* (1993) |
| Cucurbitaceae | *Lagenaria siceraria* (Molina) Standl. | X | N, S | Diurnal Lepidoptera and *Apis mellifera* | VO | Sphingidae were primary pollinators of *L. siceraria* | Morimoto *et al.* (2004) |
| Dipterocarpaceae | *Dipterocarpus obtusifolius* Teijsm. ex Miq. | N | N, S | Various diurnal Lepidoptera | P, VO |  | Ghazoul (1997) |
|  | *Dipterocarpus pachyphyllus* Meijer | X | G | None | VO |  | Harrison *et al.* (2005) |
| Ebenaceae | *Diospyros burchellii* Hiern. | U | X | None | I, VO | Not Sphingidae | Oliveira *et al.* (2004) |
| Ericaceae | *Dracophyllum ramosum* Pancher ex Brongn. & Gris | X | X | None | VO |  | Kato and Kawakita (2004) |
|  | *Erica* spp. | U | G, N | *–* | P | Pollen of *Erica cinerea* L. and *E. tetralix* L. not distinguished | Devoto *et al.* (2011) |
|  | *Rhododendron occidentale* (Torr. & A. Gray) | E | S | Diurnal Lepidoptera | C, VO |  | Grant (1983) |
|  | *Vaccinium angustifolium* Aiton | N | G, N, P, S, X | Various bees (Hymenoptera) | P |  | Cutler *et al.* (2012), Manning and Cutler (2013) |
| Escalloniaceae | *Escallonia myrtoidea* Bertero ex DC. | U | G | Various Hymenoptera, diurnal Lepidoptera, Diptera and Coleoptera | VO |  | Valdivia and Niemeyer (2006) |
| Euphorbiaceae | *Cnidoscolus texanus* (Müll.Arg.) Small | X | S | – | P, VO |  | Perkins *et al.* (1975) |
|  | *Croton dichogamus* Pax | E | S | – | VO |  | Martins and Johnson (2013) |
|  | *Croton megalocarpus* Hutch. | E | S | – | VO |  | Martins and Johnson (2013) |
|  | *Mallotus barbatus* Müll.Arg. | X | S | Hymenoptera, diurnal Lepidoptera, Diptera | VO |  | Kato *et al.* (2008) |
| Fabaceae | *Bauhinia aculeata* L. | X | N, S | Various Hymenoptera, diurnal Lepidoptera, Coleoptera and Trochilidae | P, VO |  | Hokche and Ramirez (1990) |
|  | *Bauhinia forficata* Link | I | S | None | C, P, VO | *M. sexta* is the exclusive pollinator of *B. forficata* | Neto (2013) |
|  | *Browneopsis disepala* (Little) Klitg. | U | X | Chiroptera | VO | Chiroptera are more efficient pollinators | Knudsen and Klitgaard (1998) |
|  | *Caesalpinia gilliesii* (Hook.) D. Dietr. | I | S | None | I, P, VO |  | Cocucci *et al.* (1992), Moré *et al.* (2006) |
|  | *Dalea pinnata* (J.F.Gmel.) Barneby | U | X | *–* | P, VO |  | Atwater (2013) |
|  | *Inga sessilis* (Vell.) Mart. | X | S | Birds (Aves) and Chiroptera | D, VO |  | Amorim *et al.* (2013) |
|  | *Inga* spp. | X | E, G, N, P, S, U | Trochilidae and diurnal Lepidoptera | VO |  | Koptur (1983) |
|  | *Lathyrus aphaca* L. | U | X | *–* | P, VO |  | Banza (2011) |
|  | *Melilotus indicus* (L.) All. | U | X | *–* | P, VO |  | Banza (2011) |
|  | *Scorpiurus muricatus* L. | U | X | *–* | P, VO |  | Banza (2011) |
|  | *Trifolium* spp. | U | X | *–* | P, VO |  | Banza (2011) |
|  | *Zapoteca* spp. | I | G, N, P | None | C, VO | All *Zapoteca* species are moth-pollinated | Hernández (1989) |
| Geraniaceae | *Erodium malacoides* (L.) L’Hér. | U | X | *–* | P, VO |  | Banza (2011) |
| Gesneriaceae | Various | N | X | Primarily Trochilidae and Chiroptera | C, VO, VR | Several pollination syndromes exist among this family, including moth pollination | Martén-Rodríguez *et al.* (2009) |
| Gnetaceae | *Gnetum gnemon* Linné var. *tenerum* Markgraf | X | G, P | None | P, VO |  | Kato *et al.* (1995) |
| Hyacinthaceae | *Dipcadi brevifolium* (Thunb.) Fourc. | O | N | None | P, VO | *Cornutiplusia circumflexa* L. | Manning *et al.* (2012) |
| Hypericaceae | *Hypericum pulchrum* L. | U | N | *–* | P | *Diachrysia chrysistis* L. | Devoto *et al.* (2011) |
| Iridaceae | *Gladiolus candidus* (Rendle) Goldblatt | E | S | – | VO |  | Martins and Johnson (2013) |
|  | *Gladiolus longicollis* Baker | E | S | None | P | Primarily *Agrius convolvuli* L. | Alexandersson and Johnson (2002) |
|  | *Gladiolus* spp. | U | N, S, X | Various | C, P, VO | Moth pollination has evolved six times independently within this genus | Goldblatt and Manning (2002) |
|  | Various | U | G, N, S, X | Various | I, VO | Moth pollination strategies for both hovering and settling moths within this family | Goldblatt and Manning (2006) |
| Lamiaceae | *Oxera neriifolia* (Montrouz.) Beauvis. | X | S | None | VO |  | Kato and Kawakita (2004) |
|  | *Plectranthus pubescens* Baker | E | S | – | VO |  | Martins and Johnson (2013) |
| Lecythidaceae | *Napoleonaea vogelii* Hook. & Planch. | X | Gl | Various Thysanoptera, Coleoptera and Hymenoptera | P, VO |  | Frame and Durou (2001) |
| Lentibulariaceae | *Utricularia graminifolia* Vahl | X | N, P, S, U | Various Hymenoptera and diurnal Lepidoptera | D, VO |  | Hobbhahn *et al.* (2006) |
| Liliaceae | *Lilium auratum* Lindl. | X | S, X | *Papilio bianor* Cramer (Lepidoptera: Papilionidae) | U |  | Morinaga *et al.* (2009) |
|  | *Lilium formosanum* Wallace | E | S, X | *Cyrtothyrea marginalis* Swartz (Coleoptera) | P, S, VO | Primarily *Agrius convolvuli* | Rodger *et al.* (2010) |
|  | *Lilium japonicum* Thunb. var. *japonicum* | I | G, N, P, S | None | VR |  | Yokota and Yahara (2012) |
|  | *Lilium martagon* L. | E | S | None | C, VO | Only Sphingidae considered as potential pollinators | Brantjes and Bos (1980) |
| Linaceae | *Linum tenue* Desf. | U | X | *–* | P |  | Banza (2011) |
| Loasaceae | *Mentzelia laevicaulis* (Douglas) Torr. & A. Gray | E | S | Hymenoptera | VO |  | Grant (1983) |
| Loganiaceae | *Antonia ovata* Pohl. | U | X | None | I, VO | Not Sphingidae | Oliveira *et al.* (2004) |
|  | *Strychnos pseudoquina* A. St.-Hil. | U | X | None | I, VO | Not Sphingidae | Oliveira *et al.* (2004), Martins and Batalha (2006) |
| Malvaceae | *Luehea candida* (Moc. & Sessé ex DC.) Mart. | I | N, P, S, U | None | VO |  | Haber and Frankie (1982) |
|  | *Tilia* spp. | X | Ct, E, G, N, P, Se, S | Hymenoptera and Diptera | P, VO |  | Anderson (1976) |
| Meliaceae | *Turraea mombassana* C. DC. | E | S | – | VO |  | Martins and Johnson (2013) |
| Myrtaceae | *Syzygium fastigiatum* (Blume) Merr. & L.M.Perry | X | Ct | Diurnal Lepidoptera, Hymenoptera, Coleoptera, | VO | Primarily Coleoptera | Kato *et al.* (2008) |
|  | *Syzygium tierneyanum* (F. Muell.) T.G. Hartley & L.M. Perry | I | S, U | Aves, diurnal Lepidoptera, Hymenoptera, Diptera and Chiroptera | C, VO |  | Hopper (1980) |
| Nepenthaceae | *Nepenthes vieillardii* Hook. | X | X | Coleoptera | VO |  | Kato and Kawakita (2004) |
| Nyctaginaceae | *Abronia ammophila* Greene | X | N, S | Diurnal Lepidoptera and bumblebees (Hymenoptera: Apidae) | P, VO | Noctuidae were most abundant pollinators | Saunders and Sipes (2006) |
|  | *Abronia macrocarpa* L.A. Galloway | X | N, S | None | P, VO |  | Williamson *et al.* (1994) |
|  | *Mirabilis jalapa* L. | I | S | None | C, D, VO | *Erinnyis ello* L. and *Hyles lineata* | Martinez del Rio and Burquez (1986) |
|  | *Mirabilis longiflora* L. | E | N, S | *Apis mellifera* (Hymenoptera: Apidae) | VO | *Manduca quinquemaculata* is the most important pollinator | Grant and Grant (1983a) |
|  | *Mirabilis multiflora* (Torr.) A. Gray | E | S | None | VF, VO | *Hyles lineata* | Hodges (1995) |
| Oleaceae | *Jasminum coarctatum* Roxb. | X | S | None | VO |  | Kato *et al.* (2008) |
|  | *Jasminum fluminense* Vell. | E | S | – | VO |  | Martins and Johnson (2013) |
|  | *Jasminum grandiflorum* subsp. *floribundum* (R.Br. ex Fresen.) P.S.Green | E | S | – | VO |  | Martins and Johnson (2013) |
| Onagraceae | *Calylophus hartwegii* subsp. *filifolia* (Eastw.) Towner & Raven | I | N, P, S | Hymenoptera | P, VO | Moths account for 65% of flower visitors | Clinebell *et al.* (2004) |
|  | *Gaura coccinea* Nutt. ex Pursh | O | G, N, P, S, X | Negligible | P, VO | Moths carry 99% of gross pollen load | Clinebell *et al.* (2004) |
|  | *Gaura villosa* Torr. subsp. *villosa* | O | E, G, N, P, S, X | Neuroptera; Hymenoptera | P, VO | Moths account for 63% of flower visitors | Clinebell *et al.* (2004) |
|  | *Oenothera biennis* L. | E | S | None | C, VO |  | Graham (2010) |
|  | *Oenothera drummondii* Hook. | I | S | None | P, VF | Not the focal species of this study | Eisikowitch and Galil (1971) |
|  | *Oenothera grandiflora* L’Hér. | E | N, S | None | C, VO |  | Graham (2010) |
|  | *Oenothera macrocarpa* Nutt. | E | S | None | VO |  | Moody-Weis and Heywood (2001) |
|  | *Oenothera rhombipetala* Nutt. ex Torr. & A. Gray | N | N, S |  | VO | Anecdotal evidence only | Clinebell *et al.* (2004) |
| Orchidaceae | *Aerangis brachycarpa* (A. Rich.) Durand & Schinz | E | S | None | VO |  | Martins and Johnson (2007, 2013) |
|  | *Aerangis confusa* J. Stewart | E | S | None | VO |  | Martins and Johnson (2007) |
|  | *Aerangis ellisii* (B.S. Williams) Schltr. | I | S | None | I, S | *Agrius convolvuli* and *Panogena lingens* Butler | Nilsson and Rabakonandrianina (1988) |
|  | *Aerangis kotschyana* (Rchb.f.) Schltr. | E | S | None | VO |  | Martins and Johnson (2007) |
|  | *Aerangis thomsonii* (Rolfe) Schltr. | E | S | None | VO |  | Martins and Johnson (2007) |
|  | *Angraecum arachnites* Schltr. | E | S | None | VO | *Panogena lingens* is the exclusive pollinator of *A. arachnites* | Nilsson *et al.* (1985) |
|  | *Angraecum compactum* Schltr. | E | S | None | P, VO, VR | *Coelonia solani* Boisduval, *Panogena lingens*, and *Xanthopan morganii praedicta* Rothschild and Jordan | Wasserthal (1997) |
|  | *Angraecum sesquipedale* Thouars | E | S | None | P, VO, VR | *Xanthopan morganii praedicta* | Wasserthal (1997) |
|  | *Angraecum sororium* Schltr. | E | S | None | P, VO, VR | *Coelonia solani* | Wasserthal (1997) |
|  | *Bonatea speciosa* (L.f.) Willd. | I | S | None | P, VO | *Theretra capensis* L. and *Hyles livornica* Esper. | Johnson and Liltved (1997) |
|  | *Brachycorythis helferi* (Rchb.f.) Summerh. | X | X | None | VO |  | Kato *et al.* (2008) |
|  | *Disa cooperi* Rchb. f. | I | S | None | P, VO | *Basiothia schenki* Moschler is the exclusive pollinator of *D. cooperi* | Johnson (1995a) |
|  | *Disa ophrydea* (Lindl.) Bolus | N | X | None | P, VO |  | Johnson (1995b) |
|  | *Gymnadenia conopsea* (L.) R.Br. | U | N, S | Diurnal Lepidoptera | P, VO |  | Huber *et al.* (2005) |
|  | *Gymnadenia odoratissima* (L.) Rich. | U | G, Pt, P, T | Diurnal Lepidoptera | P, VO |  | Huber *et al.* (2005) |
|  | *Habenaria decaryana* H. Perrier | O | G, N | None | P |  | Nilsson and Jonsson (1985) |
|  | *Habenaria gourlieana* Gillies ex Lindl. | I | S | None | P | *Agrius cingulata* and *Manduca sexta* | Singer and Cocucci (1997) |
|  | *Habenaria hieronymi*  Kraenzl. | O | N | None | P | *Rachiplusia nu* Guenée | Singer and Cocucci (1997) |
|  | *Habenaria johannensis* Bard. Rodr. | U | S | None | VO, VR |  | Pedron *et al.* (2012) |
|  | *Habenaria macronectar* (Vell.) Heohne | U | S | None | VO, VR |  | Pedron *et al.* (2012) |
|  | *Habenaria megapotamensis* Hoehne | U | S | None | VO, VR |  | Pedron *et al.* (2012) |
|  | *Habenaria montevidensis* Spreng. | O | X | None | I |  | Singer and Cocucci (1997) |
|  | *Habenaria parviflora* Lindl. | U | P | Various Diptera | VO |  | Singer (2001) |
|  | *Habenaria pumila* Poepp. | O | X | None | S |  | Singer and Cocucci (1997) |
|  | *Habenaria rupicola* Barb.Rodr. | O | X | None | S |  | Singer and Cocucci (1997) |
|  | *Mystacidium venosum* Harv. Ex Rolfe | E | S | None | P, VO |  | Luyt and Johnson (2001) |
|  | *Pecteilis susannae* (L.) Raf. | X | X | None | VO |  | Kato *et al.* (2008) |
|  | *Platanthera bifolia* L. Rich | X | X | None | I | Highly specialised to pollination by moths | Nilsson (1983) |
|  | *Platanthera blephariglottis* (Willd.) Lindl. | X | Se, S | None | P, VO |  | Smith and Snow (1976) |
|  | *Platanthera chlorantha* (Custer) Reichb. | X | X | None | I | Highly specialised to pollination by moths | Nilsson (1983) |
|  | *Platanthera lacera* (Michx.) G. Don | I | N | None | VO | *Anagrapha falcifera* Kirby and *Allagrapha aerea* Hübner | Little *et al.* (2005) |
|  | *Platanthera leucophaea* (Nutt.) Lindl. | E | S | None | I, P, VO |  | Bowles (1983) |
|  | *Platanthera metabifolia* subsp. *extremiorientalis* (Nevski) Soó | U | N, S | None | P |  | Inoue (1986) |
|  | *Platanthera obtusata* (Banks ex Pursh) Lindl. | O | G, P | Diptera | P, VO |  | Voss and Riefner (1983) |
|  | *Platanthera praeclara* Sheviak & M. L. Bowles | E | S | None | P | *Hyles gallii* (Rottenburg) and *Sphinx drupiferarum* J. E. Smith | Westwood and Borkowsky (2004) |
|  | *Platanthera stricta* Lindl. | X | G, Pr | *Bombus* spp.; various Empididae (Diptera) | P, VO | *Eustroma fasciata* B. and McD. (Geometridae) and a previously undescribed *Greya* sp. (Prodoxidae) | Patt *et al.* (1989) |
|  | *Prescottia plantaginea* Lindl. | N | P | None | VO |  | Singer and Sazima (2001) |
|  | *Prescottia stachyodes* (Sw.) Lindl. | N | P | None | VO |  | Singer and Sazima (2001) |
|  | *Pseudorchis albida* (L.) Á. Löve & D. Löve | N | Pt, P | None | P, VO |  | Jersáková *et al.* (2011) |
|  | *Rangaeris amaniensis* (Kraenzl.) Summerh. | E | S | None | VO |  | Martins and Johnson (2007, 2013) |
|  | *Satyrium hallackii* subsp. *ocellatum* (Bolus) A. V. Hall | X | S | Long-tongued Diptera | P, VO | A short-spurred form, *S. hallackii* subsp. *hallackii*, is primarily pollinated by bees (Hymenoptera) | Johnson (1997) |
|  | *Satyrium longicauda* Lindl. (Orchidaceae) | X | N, S | None | VO |  | Jersáková and Johnson (2007), Johnson *et al.* (2009) |
|  | *Sauroglossum nitidum* (Vell.) Schltr. | U | N | None | VO |  | Singer (2002) |
|  | *Tipularia discolor* (Pursh) Nutt. | N | N | None | VO | *Mythimna unipuncta* Haworth | Whigham and McWethy (1980) |
|  | Various Angraecinae | E | S | *–* | P | Various Sphingidae were flower visitors of the study species, but only *Panogena lingens* was found to carry pollen | Nilsson *et al.* (1987) |
| Orobanchaceae | *Cycnium ajugifolium* Engl. | E | S | – | VO |  | Martins and Johnson (2013) |
|  | *Cycnium tubulosum* (L.f.) Engl. | E | S | – | VO |  | Martins and Johnson (2013) |
| Passifloraceae | *Passiflora capsularis* L. | X | X | *–* | I |  | Koschnitzke and Sazima (1997) |
|  | *Passiflora mooreana* Hook. f. | E | S | Various Hymenoptera | I, VO | *Erinnyis ello* | Garcia and Hoc (1998) |
| Phrymaceae | *Mimulus aurantiacus* Curtis | X | S | Trochilidae | VO | Yellow-flowered inland race of *M. aurantiacus* appears to be evolved to promote moth pollination by *Hyles lineata* | Streisfeld and Kohn (2007) |
| Phyllanthaceae | *Breynia fruticosa* (L.) Müll.Arg. | X | Gr | None | VO |  | Kato *et al.* (2008) |
|  | *Glochidion caledonicum* Müll.Arg. | X | Gr | None | VO |  | Kato and Kawakita (2004) |
|  | *Glochidion rubrum* Blume | X | Gr | None | VO |  | Kato *et al.* (2008) |
|  | *Phyllanthus aeneus* Baill. | X | Gr | None | VO |  | Kato and Kawakita (2004) |
|  | *Phyllanthus bourgeoisie* Baill. | X | Gr | None | VO |  | Kato and Kawakita (2004) |
|  | *Phyllanthus cochinchinensis* Spreng. | O | Ge | None | P, VO | Obligate pollinating seed parasite mutualism | Luo *et al.* (2011) |
|  | *Phyllanthus mangenotii* M.Schmid | X | Gr | None | VO |  | Kato and Kawakita (2004) |
|  | *Phyllanthus reticulates* Poir. | X | Gr | None | VO |  | Kato *et al.* (2008) |
|  | *Phyllanthus rheophyticus* M. G. Gilbert & P. T. Li | O | Ge | None | P, VO | Obligate pollinating seed parasite mutualism | Luo *et al.* (2011) |
|  | *Phyllanthus tritepalus* M.Schmid | X | Gr | None | VO |  | Kato and Kawakita (2004) |
| Plantaginaceae | *Plantago* spp. | U | X | *–* | P, VO |  | Banza (2011) |
| Polemoniaceae | *Navarretia brandegeei* (A. Gray) Kuntze | E | S | *Selasphorus platycercus* Swainson (Trochilidae) | P, VR | *Hyles lineata* and *H. gallii* | Kulbaba and Worley (2012) |
| Polygonaceae | *Eriogonum tomentosum* Michx. | U | X | *–* | P, VO |  | Atwater (2013) |
| Primulaceae | *Anagallis arvensis* L. | U | X | *–* | P, VO |  | Banza (2011) |
|  | *Primula vulgaris* Huds. | U | X | None | E | Pollination by a moth community demonstrated but which species were involved was not established | Boyd *et al.* (1990) |
| Proteaceae | *Grevillea exul* Lindl. | X | S, X | Coleoptera | VO |  | Kato and Kawakita (2004) |
|  | *Roupala Montana* Aubl. | U | X | None | I, VO | Not Sphingidae | Oliveira *et al.* (2004), Martins and Batalha (2006) |
| Ranunculaceae | *Aquilegia caerulea* E. James | E | S | *Bombus* spp. | P, VO | *Hyles lineata* | Miller (1978) |
|  | *Aquilegia chrysantha* A. Gray | E | S | None | P, VO | *Eumorpha achemon* Drury appears to be the most important pollinator. Other species of Sphingidae, including *Sphinx chersis* Hübner and *S. asella* Rothschild & Jordan, may also contribute | Miller (1985) |
|  | *Aquilegia pubescens* Coville | I | S | Various Hymenoptera and Trochilidae | VO |  | Fulton and Hodges (1999) |
|  | *Delphinium leroyi* Franch. ex Huth | E | S | None | P, VO | *Hippotion celerio* L. and possibly other Sphingidae | Johnson (2001) |
|  | *Nigella damascena* L. | U | X | *–* | P, VO |  | Banza (2011) |
| Rhamnaceae | *Colubrina asiatica* (L.) Brongn. | X | X | Neuroptera, Coleoptera, Hymenoptera, Diptera | VO |  | Kato and Kawakita (2004) |
| Rosaceae | *Prunus* spp. | U | X | *–* | P, VO |  | Banza (2011) |
|  | *Rubus chamaemorus* L. | N | X | Various Hymenoptera and Diptera | E | Nocturnal visitors, possibly moths, were capable pollinators, but less effective than diurnal pollinators | Pelletier *et al.* (2001) |
| Rubiaceae | *Alibertia edulis* (Rich.) A.Rich. ex DC. | U | X | None | I, VO | Not Sphingidae | Oliveira *et al.* (2004) |
|  | *Catunaregam spinosa* (Thunb.) Tirveng. | X | S | Diurnal Lepidoptera, Hymenoptera, Coleoptera | VO |  | Kato *et al.* (2008) |
|  | *Chomelia ribesioides* Benth. ex A.Gray | U | X | – | I |  | Martins and Batalha (2006) |
|  | *Conostomium quadrangulare* (Rendle) Cufod. | E | S | – | VO |  | Martins and Johnson (2013) |
|  | *Faramea hyacinthina* Mart. | U | N, S | Various Hymenoptera | VO |  | Maruyama *et al.* (2010) |
|  | *Ferdinandusa elliptica* Pohl. | U | S | None | I, VO |  | Oliveira *et al.* (2004) |
|  | *Meyna pubescens* (Kurz) Robyns | X | S | Diurnal Lepidoptera, Hymenoptera | VO |  | Kato *et al.* (2008) |
|  | *Mitragyna rotundifolia* (Roxb.) Kuntze | X | Ct | Hymenoptera, diurnal Lepidoptera, Hemiptera, Coleoptera | VO |  | Kato *et al.* (2008) |
|  | *Morinda citrifolia* L. | X | S | None | VO |  | Kato and Kawakita (2004) |
|  | *Ophiorrhiza grandiflora* Wight | X | S | None | C, VO |  | Devy and Davidar (2003, 2006) |
|  | *Oxyanthus pyriformis* subsp. *pyriformis* (Hochst.) Skeels | E | S | None | P, VO | *Coelonia mauritii* Butler, *Nephele accentifera* de Beauvois, and possibly others | Johnson (2004) |
|  | *Palicourea faxlucens* (Lorence & Dwyer) | X | S | None | U |  | Pérez-Nasser *et al.* (1993) |
|  | *Pavetta abyssinica* Fresen. | E | S | – | VO |  | Martins and Johnson (2013) |
|  | *Pentanisia ouranogyne* S.Moore | E | S | – | VO |  | Martins and Johnson (2013) |
|  | *Sherardia arvensis* L. | U | X | *–* | P, VO |  | Banza (2011) |
|  | *Tocoyena formosa* (Cham. & Schltdl.) K.Schum. | U | S | None | I, VO |  | Oliveira *et al.* (2004), Martins and Batalha (2006) |
| Rutaceae | *Galipea jasminiflora* (A. St.-Hil.) Engl. | X | G | Diurnal Lepidoptera | VO |  | Piedade and Ranga (1993) |
| Santalaceae | *Exocarpos neocaledonicus* Schltr. & Pilg. | X | X | Neuroptera | VO |  | Kato and Kawakita (2004) |
|  | *Exocarpos phyllanthoides* Endl. | X | X | None | VO |  | Kato and Kawakita (2004) |
| Sapotaceae | *Pouteria ramiflora* (Mart.) Radlk. | U | X | – | I |  | Martins and Batalha (2006) |
|  | *Pouteria torta* (Mart.) Radlk. | U | X | – | I |  | Martins and Batalha (2006) |
| Saxifragaceae | *Heuchera cylindrica* Douglas | O | Pr | – | E, P, VO | *Greya enchrysa* Davis & Pellmyr. Pollinating seed parasite mutualism | Pellmyr *et al.* (1996) |
|  | *Lithophragma parviflorum* (Hook.) Nutt. | O | Pr | – | E, VO | *Greya politella* Walsingham. Pollinating seed parasite mutualism | Thompson and Pellmyr (1992) |
|  | *Mitella stauropetala* Piper | O | Pr | – | E, P, VO | *Greya mitellae* Davis & Pellmyr. Pollinating seed parasite mutualism | Pellmyr *et al.* (1996) |
| Scrophulariaceae | *Bellardia trixago* All. | U | X | *–* | P, VO |  | Banza (2011) |
|  | *Buddleja davidii* Franch. | U | G, N, P, T | Various, including butterflies (Lepidoptera) and *Apis mellifera* | VT |  | Guédot *et al.* (2008) |
| Solanaceae | *Datura ferox* L. | N | S | Various Coleoptera, *Apis mellifera* | VO |  | Torres *et al.* (2013) |
|  | *Datura innoxia* Mill. | E | S | None | VO | *Manduca sexta,* *M. quinquemaculata,* and *Hyles lineata* | Grant and Grant (1983b) |
|  | *Datura stramonium* L. | E | S | – | VO |  | Martins and Johnson (2013) |
|  | *Datura wrightii* Regel | I | S | None | P | *Manduca sexta* (Sphingidae) | Alarcón *et al.* (2008), Bronstein *et al.* (2009) |
|  | *Nicotiana attenuata* Torr. ex S.Watson | E | S | – | VO |  | Grant (1983) |
|  | *Petunia axillaris* (Lam.) Britton, Sterns & Poggenb. | N | S | None | VO | *Manduca* spp. | Ando *et al.* (2001) |
| Thymelaeaceae | *Aquilaria crassna* Pierre ex Lecomte | U | E, G, L, N, P, Th | Various Hymenoptera, Coleoptera and Diptera | P, VO | Moths (61 spp.) were the most species-rich and the most frequent flower visitors | Tasen *et al.* (2009) |
|  | *Diplomorpha ganpi* (Siebold & Zucc.) Nakai | O | E, G, N, P | Diurnal Lepidoptera | P, VO |  | Okamoto *et al.* (2008) |
|  | *Diplomorpha phymatoglossa* (Koidz.) Nakai | O | G, N, P | None | P, VO |  | Okamoto *et al.* (2008) |
|  | *Diplomorpha sikokiana* (Franch. & Sav.) Honda | O | G, N, No, P | Various Coleoptera, Diptera, Hymenoptera and diurnal Lepidoptera | P, VO |  | Okamoto *et al.* (2008) |
|  | *Diplomorpha trichotoma* (Thunb.) Nakai | O | G, P | Diptera | P, VO |  | Okamoto *et al.* (2008) |
|  | *Diplomorpha yakushimensis* (Makino) Masam. | O | G, P | None | P, VO |  | Okamoto *et al.* (2008) |
|  | *Struthiola ciliata* (L.) Lam. | O | N | None | P, VO | *Syngrapha circumflexa* L. and *Cucullia terensis* Felder and Rogenhofer | Makholela and Manning (2006) |
|  | *Wikstroemia indica* (L.) C.A. Mey. | X | X | None | VO |  | Kato and Kawakita (2004) |
| Urticaceae | *Urtica* spp. | U | X | *–* | P, VO |  | Banza (2011) |
| Verbenaceae | *Lantana camara* L. | E | S | None | VO |  | Kato and Kawakita (2004), Martins and Johnson (2013) |
|  | *Lippia javanica* (Burm.f.) Spreng. | E | S | – | VO |  | Martins and Johnson (2013) |
|  | *Lippia rosmarinifolia* Andersson | N | P, X | None | VO |  | Philipp *et al.* (2006) |
| Violaceae | *Viola cazorlensis* Gand. | X | S | None | VO | *Macroglossum stellatarum* L. (diurnal) | Herrera (1993) |
| Vochysiaceae | *Qualea grandiflora* Mart. | U | S | None | I, VO |  | Oliveira *et al.* (2004), Martins and Batalha (2006) |
|  | *Salvertia convallariodora* A. St.-Hil. | X | S | None | I, VO | Primarily *Erinnyis ello* | Oliveira (1996), Oliveira *et al.* (2004) |
|  | *Vochysia pyramidalis* Mart. | X | S | Various Hymenoptera | VO |  | Oliveira and Gibbs (1994) |
|  | *Vochysia thyrsoidea* Pohl | X | S | Various Hymenoptera | VO |  | Oliveira and Gibbs (1994) |
|  | *Vochysia tucanorum* Mart. | X | S | Various Hymenoptera | VO |  | Oliveira and Gibbs (1994) |
| Winteraceae | *Zygogynum baillonii* Tiegh. | X | M | None | VO |  | Kato and Kawakita (2004) |
|  | *Zygogynum* spp. | X | M | None | U | *Sabatinca* spp. | Thien *et al.* (1985) |

**Table S1.3:** Examples of moths acting as pollinating seed parasites.

| Plant family | Plant species | Moth species | Notes | Reference |
| --- | --- | --- | --- | --- |
| Asparagaceae | *Yucca* spp. | *Tegeticula* spp. and *Parategeticula* spp. (Prodoxidae) | Obligate relationship | Pellmyr *et al.* (1996) |
| Cactaceae | *Lophocereus schottii* (Engelm.) Britton & Rose | *Upiga virescens* Hulst (Pyralidae) | *U. virescens* responsible for >90% of pollination in *L. schottii* | Fleming and Holland (1998), Holland and Fleming (1999) |
| Caryophyllaceae | *Dianthus sylvestris* Wulfen | *Hadena* *compta* Denis & Schiffermüller (Noctuidae) |  | Collin *et al.* (2002) |
|  | *Silene dioica* (L.) Clairv. | *Perizoma affinitatum* Stephens (Geometridae) |  | Westerbergh (2004) |
|  | *Silene* spp. | *Hadena* spp. (Noctuidae) |  | Kephart *et al.* (2006) |
|  | *Silene stellata* (L.) W.T. Aiton | *Hadena ectypa* Morrison (Noctuidae) |  | Kula *et al.* (2013) |
| Phyllanthaceae | *Glochidion* spp. | *Epicephala* spp. (Gracillariidae) | Species-specific pollinating seed parasites exist for at least 3 species of *Glochidion*. | Kato *et al.* (2003), Hembry *et al* (2013) |
|  | *Phyllanthus cochinchinensis* Spreng. | Unknown *Deltaphora* sp. (Gelechiidae) |  | Luo *et al.* (2011) |
|  | *Phyllanthus rheophyticus* M. G. Gilbert & P. T. Li | Unknown *Deltaphora* sp. (Gelechiidae) |  | Luo *et al.* (2011) |
| Saxifragaceae | *Heuchera cylindrica* Douglas | *Greya enchrysa* Davis & Pellmyr |  | Pellmyr *et al.* (1996) |
|  | *Lithophragma parviflorum* (Hook.) Torr. & Gray | *Greya politella* Walsingham (Prodoxidae) |  | Thompson and Pellmyr (1992) |
|  | *Mitella stauropetala* Piper | *Greya mitellae* Davis & Pellmyr |  | Pellmyr *et al.* (1996) |

**Table S2:** An examination of bias towards Sphingidae in studies of moth pollination (see Table S1.2). Studies are separated by presence or absence of a stated or implied prediction of pollination by Sphingidae (sphingophily) and by type of moth-pollination: sphingophily, phalaenophily, or both. Brackets indicate percentage of studies within the relevant prediction category. In column 2, ‘wider taxa’ includes any named group at a hierarchical level above species and below family.

| Prediction of moth-pollination | No. studies | No. species or wider taxa | Studies finding Sphingidae only as pollinators (sphingophily) | Studies finding Sphingidae and other moths as pollinators | Studies finding other moths only as pollinators (phalaenophily) or unspecified |
| --- | --- | --- | --- | --- | --- |
| Explicit or implicit prediction of sphingophily | 56 | 92 | 38 (67.8%) | 15 (26.8%) | 3 (5.4%) |
| Prediction of non-Sphingidae pollination, general/unspecified moth pollination or no explicit prediction | 103 | 201 | 21 (20.4%) | 29 (28.2%) | 53 (51.4%) |

**Table S3:** Studies of moths involved in pollination by family (see Table S1.2). In column 2, ‘wider taxa’ includes any named group at a hierarchical level above species and below family.

| Family | No. species or wider taxa pollinated | No. plant families pollinated |
| --- | --- | --- |
| Cosmopterigidae | 1 | 1 |
| Crambidae | 1 | 1 |
| Ctenuchidae | 3 | 3 |
| Erebidae | 8 | 7 |
| Gelechiidae | 2 | 1 |
| Geometridae | 34 | 18 |
| Glyphipterigidae | 1 | 1 |
| Gracillariidae | 8 | 1 |
| Lasiocampidae | 2 | 2 |
| Micropterigidae | 2 | 1 |
| Noctuidae | 62 | 24 |
| Nolidae | 1 | 1 |
| Prodoxidae | 5 | 3 |
| Pterophoridae | 2 | 1 |
| Pyralidae | 32 | 15 |
| Saturniidae | 1 | 1 |
| Sesiidae | 2 | 2 |
| Sphingidae | 154 | 34 |
| Thyrididae | 1 | 1 |
| Tortricidae | 3 | 3 |
| Uranidae | 3 | 2 |

**Additional references (cited only in supplementary information)**

1. Alarcón, R., Davidowitz, G. and Bronstein, J.L. (2008) Nectar usage in a southern Arizona hawkmoth community. *Ecological Entomology*, **33**, 503–509.
2. Alexandersson, R. and Johnson, S.D. (2002) Pollinator-mediated selection on flower-tube length in a hawkmoth-pollinated *Gladiolus* (Iridaceae). *Proceedings of the Royal Society B*, **269**, 631–636.
3. Alonso-Pedano, M. and Ortega-Baes, P. (2012) Generalized and complementary pollination system in the Andean cactus *Echinopsis schickendantzii. Plant Systematics and Evolution*, **298**, 1671–1677.
4. Amorim, F.W., Galetto, L. and Sazima, M. (2013) Beyond the pollination syndrome: nectar ecology and the role of diurnal and nocturnal pollinators in the reproductive success of *Inga sessilis* (Fabaceae). *Plant Biology*, **15**, 317­–327.
5. Anderson, G.J. (1976) The pollination biology of *Tilia*. *American Journal of Botany*, **63**, 1203–1212.
6. Ando, T., Nomura, M., Tsukahara, J., Watanabe, H., Kokubun, H., Tsukamoto, T., Hashimoto, G., Marchesi, E. and Kitching, I.J. (2001) Reproductive isolation in a native population of *Petunia sensu* Jussieu (Solanaceae). *Annals of Botany*, **88**, 403–413.
7. Arizaga, S., Ezcurra, E., Peters, E., de Arellano, F.R. and Vega, E. (2000a) Pollination ecology of *Agave macroacantha* (Agavaceae) in a Mexican tropical desert. I. Floral biology and pollination mechanisms. *American Journal of Botany*, **87**, 1004–1010.
8. Arizaga, S., Ezcurra, E., Peters, E., de Arellano, F.R. and Vega, E. (2000b) Pollination ecology of *Agave macroacantha* (Agavaceae) in a Mexican tropical desert. II. The role of pollinators. *American Journal of Botany*, **87**, 1011–1017.
9. Atwater, M.M. (2013) Diversity and nectar hosts of flower-settling moths within a Florida sandhill ecosystem. *Journal of Natural History*, doi: 10.1080/00222933.2013.791944.
10. Barthlott, W., Porembski, S., Kluge, M., Hopke, J. and Schmidt, L. (1997) *Selenicereus wittii* (Cactaceae): an epiphyte adapted to Amazonian Igapó inundation forests. *Plant Systematics and Evolution*, **206**, 175–185.
11. Bowles, M.L. (1983) The tallgrass prairie orchids *Platanthera leucophaea* (Nutt.) Lindl. and *Cypripedium candidum* Muhl. ex Willd.: some aspects of their status, biology, and ecology, and implications toward management. *Natural Areas Journal*, **3**, 14–37.
12. Boyd, A.E. (2004) Breeding system of *Macromeria viridiflora* (Boraginaceae) and geographic variation in pollinator assemblages. *American Journal of Botany*, **91**, 1809–1813.
13. Boyd, M., Silvertown, J. and Tucker, C. (1990) Population ecology of heterostyle and homostyle *Primula vulgaris*: growth, survival and reproduction in field populations. *Journal of Ecology*, **78**, 799–813.
14. Brantjes, N.B.M. and Bos, J.J. (1980) Hawkmoth behaviour and flower adaptation reducing self pollination in two Liliiflorae. *New Phytologist*, **84**, 139–143.
15. Bronstein, J.L., Huxman, T., Horvath, B., Farabee, M. and Davidowitz, G. (2009) Reproductive biology of *Datura wrightii*: the benefits of a herbivorous pollinator. *Annals of Botany*, **103**, 1435–1443.
16. Clinebell, R.R.I., Crowe, A., Gregory, D.P. & Hoch, P.C. (2004) Pollination ecology of *Gaura* and *Calylophus* (Onagraceae, tribe Onagreae) in western Texas, U.S.A. *Annals of the Missouri Botanical Garden*, **91**, 369–400.
17. Cocucci, A.A., Galetto, L. and Sersic A. (1992) The floral syndrome of *Caesalpinia gilliesii* (Fabaceae – Caesalpinioideae). *Darwiniana*, **31**, 111–135.
18. Collin, C.L., Pennings, P.S., Ruefller, C., Widmer, A. & Shykoff, J.A. (2002) Natural enemies and sex: how seed predators and pathogens contribute to sex-differential reproductive success in a gynodioecious plant. *Oecologia*, **131**, 94–102.
19. Cutler, G.C., Reeh, K.W., Sproule, J.M. & Ramanaidu, K. (2012) Berry unexpected: Nocturnal pollination of lowbush blueberry. *Canadian Journal of Plant Science*, **92**, 707–711.
20. Devy, M.S. and Davidar P. (2003) Pollination systems of trees in Kakachi, a mid-elevation wet evergreen forest in Western Ghats, India. *American Journal of Botany*, **90**, 650–657.
21. Devy, M.S. and Davidar P. (2006) Breeding systems and pollination modes of understorey shrubs in a medium elevation wet evergreen forest, southern Western Ghats, India. *Current Science*, **90**, 838–842.
22. Eisikowitch, D. and Galil, J. (1971) Effect of wind on the pollination of *Pancratium maritimum* L. (Amaryllidaceae) by hawkmoths (Lepidoptera: Sphingidae). *Journal of Animal Ecology*, **40**, 673–678.
23. Erhardt, A. (1990) Pollination of *Dianthus gratianopolitanus* (Caryophyllaceae). *Plant Systematics and Evolution*, **170**, 125–132.
24. Erhardt, A. (1991) Pollination of *Dianthus superbus* L. *Flora* **185**, 99–106.
25. Fagua, J.C. and Gonzalez, V.H. (2007) Growth rates, reproductive phenology, and pollination ecology of *Espeletia grandiflora* (Asteraceae), a giant Andean caulescent rosette. *Plant Biology*, **9**, 127–135.
26. Fleming, T.H. & Holland, J.N. (1998) The evolution of obligate pollination mutualisms: senita cactus and senita moth. *Oecologia*, **114**, 368–375.
27. Frame, D. and Durou, S. (2001) Morphology and biology of *Napoleonaea vogelii* (Lecythidaceae) flowers in relation to the natural history of insect visitors. *Biotropica*, **33**, 458–471.
28. Fulton, M. & Hodges, S.A. (1999) Floral isolation between *Aquilegia formosa* and *Aquilegia pubescens*. *Proceedings of the Royal Society B*, **266**, 2247–2252.
29. Garcia, M.T.A. and Hoc, P.S. (1998) Floral biology and reproductive system of *Passiflora mooreana* (Passifloraceae). *Darwiniana*, **35**, 9–27.
30. Gentry, A.H. (1990) *Sphingiphila* (Bignoniaceae), a new genus from the Paraguayan Chaco. *Systematic Biology*, **15**, 277–279.
31. Ghazoul, J. (1997) The pollination and breeding system of *Dipterocarpus obtusifolius* (Dipterocarpaceae) in dry deciduous forests of Thailand. *Journal of Natural History*, **31**, 901–916.
32. Goldblatt, P. & Manning, J.C. (2002) Evidence for moth and butterfly pollination in *Gladiolus* (Iridaceae – Crocoideae). *Annals of the Missouri Botanical Garden*, **89**, 110–124.
33. Goldblatt, P. and Manning, J.C. (2006) Radiation of pollination systems in the Iridaceae of sub-Saharan Africa. *Annals of Botany*, **97**, 317–344.
34. Graham, S.P. (2010) Visitors to southeastern hawkmoth flowers. *Southeastern Naturalist*, **9**, 413–426.
35. Grant, V. (1983) The systematic and geographical distribution of hawkmoth flowers in the temperate North American flora. *Botanical Gazette*, **144**, 439–449.
36. Grant, V. & Grant, K.A. (1983a) Hawkmoth pollination of *Mirabilis longiflora* (Nyctaginaceae). *Proceedings of the National Academy of Sciences*, **80**, 1298–1299.
37. Grant, V. & Grant, K.A. (1983b) Behavior of hawkmoths on flowers of *Datura meteloides. Botanical Gazette*, **144**, 280–284.
38. Gribel, R. and Hay, J.D. (1993) Pollination ecology of *Caryocar brasiliense* (Caryocaraceae) in central Brazil cerrado vegetation. *Journal of Tropical Ecology*, **9**, 199–211.
39. Groman, J.D. and Pellmyr, O. (1999) The pollination biology of *Manfreda virginica* (Agavaceae): relative contribution of diurnal and nocturnal visitors. *Oikos*, **87**, 373–381.
40. Guédot, C., Landolt, P.J. & Smithhisler, C.L. (2008) Odorants of the flowers of butterfly bush, *Buddleja davidii*, as possible attractants of pest species of moths. *Florida Entomologist*, **91**, 576–582.
41. Haber, W.A. and Frankie, G.W. (1982) Pollination of *Leuhea* (Tiliaceae) in Costa Rican deciduous forest. *Ecology*, **63**, 1740–1750.
42. Haber, W.A. & Frankie, G.W. (1989) A tropical hawkmoth community: Costa Rican dry forest Sphingidae. *Biotropica*, **21**, 155–172.
43. Harrison, R.D., Nagamitsu, T., Momose, K. and Inoue, T. (2005) Flowering phenology and pollination of *Dipterocarpus* (Dipterocarpaceae) in Borneo. *Malayan Nature Journal*, **57**, 67–80.
44. Hembry, D.H., Kawakita, A., Gurr, N.E., Schmaedick, M.A., Baldwin, B.G. and Gillespie, R.G. (2013) Non-congruent colonizations and diversification in a coevolving pollination mutualism on oceanic islands. *Proceedings of the Royal Society B*, **280**, 20130361.
45. Hernández, H.M. (1989) Systematics of *Zapoteca* (Leguminosae). *Annals of Missouri Botanical Garden*, **76**, 781–862.
46. Herrera, C.M. (1993) Selection on floral morphology and environmental determinants of fecundity in a hawk moth-pollinated violet. *Ecological Monographs*, **63**, 251–275.
47. Hobbhahn, N., Küchmeister, H., and Porembski, S. (2006) Pollination biology of mass flowering terrestrial *Utricularia* species (Lentibulariaceae) in the Indian Western Ghats. *Plant Biology*, **8**, 791–804.
48. Hodges, S.A. (1995) The influence of nectar production on hawkmoth behaviour, self pollination, and seed production in *Mirabilis multiflora* (Nyctaginaceae). *American Journal of Botany*, **82**, 197–204.
49. Hokche, O. and Ramirez, N. (1990) Pollination ecology of seven species of *Bauhinia* L. (Leguminosae: Caesalpinioideae). *Annals of the Missouri Botanical Garden*, **77**, 559–572.
50. Holland, J.N. & Fleming, T.H. (1999) Mutualistic interactions between *Upiga virescens* (Pyralidae), a pollinating seed-consumer, and *Lophocereus schottii* (Cactaceae). *Ecology*, **80**, 2074–2084.
51. Holmes, D.S. (2005) Sexual reproduction in British populations of *Adoxa moschatellina* L. *Watsonia*, **25**, 265–273.
52. Hopper, S.D. (1980) Pollination of the rain-forest tree *Syzygium tierneyanum* (Myrtaceae) at Kuranda, Northern Queensland. *Australian Journal of Botany*, **28**, 223–237.
53. Howell, G. and Prakash, N. (1990) Embryology and reproductive ecology of the Darling Lily, *Crinum flaccidum* Herbert. *Australian Journal of Botany*, **38**, 433–444.
54. Huber, F.K., Kaiser, R., Sauter, W. and Schiestl, F.P. (2005) Floral scent emission and pollinator attraction in two species of *Gymnadenia* (Orchidaceae). *Oecologia*, **142**, 564–575.
55. Inoue, K. (1986) Different effects of sphingid and noctuid moths on the fecundity of *Platanthera metabifolia* (Orchidaceae) in Hokkaido. *Ecological Research*, **1**, 25–36.
56. Jennersten, O. (1988) Pollination of *Viscaria vulgaris* (Caryophyllaceae): the contributions of diurnal and nocturnal insects to seed set and seed predation. *Oikos*, **52**, 319–327.
57. Jersáková, J. and Johnson, S.D. (2007) Protandry promotes male pollination success in a moth-pollinated orchid. *Functional Ecology*, **21**, 496–504.
58. Jersáková, J., Malinová, T., Jeřábková, K. and Dötterl, S. (2011) Biological flora of the British Isles: *Pseudorchis albida* (L.) Á. & D. Löve. *Journal of Ecology*, **99**, 1282–1298.
59. Johnson, S.D. (1995a) Observations of hawkmoth pollination in the South African orchid *Disa cooperi*. *Nordic Journal of Botany*, **15**, 121–125.
60. Johnson, S.D. (1995b) Moth pollination of the cryptic cape orchid *Monadenia ophrydea*. *Flora*, **190**, 105–108.
61. Johnson, S.D. (1997) Pollination ecotypes of *Satyrium hallackii* (Orchidaceae) in South Africa. *Botanical Journal of the Linnean Society*, **123**, 225–235.
62. Johnson, S.D. (2001) Hawkmoth pollination and hybridization in *Delphinium leroyi* (Ranunculaceae) on the Nyika Plateau, Malawi. *Nordic Journal of Botany*, **21**, 599–605.
63. Johnson, S.D. (2004) An overview of plant-pollinator relationships in southern Africa. *International Journal of Tropical Insect Science*, **24**, 45–54.
64. Johnson, S.D. and Liltved, W.R. (1997) Hawkmoth pollination of *Bonatea speciosa* (Orchidaceae) in a South African coastal forest. *Nordic Journal of Botany*, **17**, 5–10.
65. Johnson, S.D., Ellis, A., Carrick, P., Swift, A., Horner, N., Vanrensburg, S.J. and Bond, W.J. (1993) Moth pollination and rhythms of advertisement and reward in *Crassula fascicularis* (Crassulaceae). *South African Journal of Botany*, **59**, 511–513.
66. Johnson, S.D., Torninger, E. and Agren, J. (2009) Relationships between population size and pollen fates in a moth-pollinated orchid. *Biology Letters*, **5**, 282–285.
67. Kato, M., Takimura, A. & Kawakita, A. (2003) An obligate pollination mutualism and reciprocal diversification in the tree genus *Glochidion* (Euphorbiaceae). *Proceedings of the National Academy of Sciences*, **100**, 5264–5267.
68. Kato, M., Kosaka, Y., Kawakita, A., Okuyama, Y., Kobayashi, C., Phimminith, T. & Thongphan, D. (2008) Plant-pollinator interactions in tropical monsoon forests in Southeast Asia. *American Journal of Botany*, **95**, 1375–1394.
69. Kephart, S., Reynolds, R.J., Rutter, M.T., Fenster, C.B. & Dudash, M.R. (2006) Pollination and seed predation by moths on *Silene* and allied Caryophyllaceae: evaluating a model system to study the evolution of mutualisms. *New Phytologist*, **169**, 667–680.
70. Knudsen, J.T. and Klitgaard, B.B. (1998) Floral scent and pollination in *Browneopsis disepala* (Leguminosae: Caesalpinioideae) in western Ecuador. *Brittonia*, **50**, 174–182.
71. Koptur, S. (1983) Flowering phenology and floral biology of *Inga* (Fabaceae: Mimosoideae). *Systematic Botany*, **8**, 354–368.
72. Koschnitzke, C. and Sazima, M. (1997) Floral biology of five species of *Passiflora* L. (Passifloraceae) in a semideciduous forest. *Revista Brasileira de Botanica*, **20**, 119–126.
73. Kula, A.A.R., Dudash, M.R. and Fenster, C.B. (2013) Choices and consequences of oviposition by a pollinating seed predator, *Hadena ectypa* (Noctuidae), on its host plant, *Silene stellata* (Caryophyllaceae). *American Journal of Botany*, **100**, 1148–1154.
74. Kulbaba, M.W. and Worley, A.C. (2012) Selection on floral design in *Polemonium brandegeei* (Polemoniaceae): female and male fitness under hawkmoth pollination. *Evolution*, **66**, 1344–1359.
75. Lin, S. and Bernardello, G. (1999) Flower structure and reproductive biology in *Aspidosperma quebracho-blanco* (Apocynaceae), a tree pollinated by deceit. *International Journal of Plant Sciences*, **160**, 869–878.
76. Little, K.J., Dieringer, G. and Romano, M. (2005) Pollination ecology, genetic diversity and selection on nectar spur length in *Platanthera lacera* (Orchidaceae). *Plant Species Biology*, **20**, 183–190.
77. Luo, S., Li, Y., Chen, S., Zhang, D. & Renner, S.S. (2011) Gelechiidae moths are capable of chemically dissolving the pollen of their host plants: First documented sporopollenin breakdown by an animal. *PLOS ONE*, **6**, e19219.
78. Luyt, R. and Johnson, S.D. (2001) Hawkmoth pollination of the African epiphytic orchid *Mystacidium venosum*, with special reference to flower and pollen longevity. *Plant Systematics and Evolution*, **228**, 49–62.
79. Makholela, T. & Manning, J.C. (2006) First report of moth pollination in *Struthiola ciliata* (Thymelaeaceae) in southern Africa. *South African Journal of Botany*, **72**, 597–603.
80. Manning, P. and Cutler, G.C. (2013) Potential nocturnal insect pollinators of lowbush blueberry. *Journal of the Acadian Entomological Society*, **9**, 1–3.
81. Manning, J.C., Goldblatt, P., Parker, E. and Kaiser, R. (2012) First record of pollination in the Afro-Eurasian *Dipcadi* Medik. (Hyacinthaceae): pollination of *D. brevifoliumi* by the owlet moth *Syngrapha circumflexa* (Noctuidae). *South African Journal of Botany*, **81**, 15–18.
82. Martén-Rodríguez, S., Almarales-Castro, A. & Fenster, C.B. (2009) Evaluation of pollination syndromes in Antillean Gesneriaceae: evidence for bat, hummingbird and generalized flowers. *Journal of Ecology*, **97**, 348–359.
83. Martinez del Rio, C. & Burquez, A. (1986) Nectar production and temperature dependent pollination in *Mirabilis jalapa* L. *Biotropica*, **18**, 28–31.
84. Martins, F.Q. and Batalha, M.A. (2006) Pollination systems and floral traits in cerrado woody species of the Upper Taquari region (central Brazil). *Brazilian Journal of Biology*, **66**, 543–552.
85. Martins, D.J. and Johnson, S.D. (2007) Hawkmoth pollination of aerangoid orchids in Kenya, with special reference to nectar sugar concentration gradients in the floral spurs. *American Journal of Botany*, **94**, 650–659.
86. Martins, D.J. and Johnson, S.D. (2013) Interactions between hawkmoths and flowering plants in East Africa: polyphagy and evolutionary specialization in an ecological context. *Biological Journal of the Linnean Society*, **110**, 199–213.
87. Maruyama, P.K., Amorim, F.W. and Oliveira P.E. (2010) Night and day service: distyly and mixed pollination system in *Faramea cyanea* (Rubiaceae). *Flora*, **205**, 818–824.
88. McMullen, C.K. (2007) Pollination biology of the Galápagos endemic, *Tournefortia rufo-sericea* (Boraginaceae). *Botanical Journal of the Linnean Society*, **153**, 21–31.
89. McMullen, C.K. (2009) Pollination biology of a night-flowering Galápagos endemic, *Ipomoea habeliana* (Convulvulaceae). *Botanical Journal of the Linnean Society*, **160**, 11–20.
90. McNeill, J. (1977) The biology of Canadian weeds. 25. *Silene alba* (Miller) E. H. L. Krause. *Canadian Journal of Plant Science*, **57**, 1103–1114.
91. Miller, R.B. (1978) The pollination ecology of *Aquilegia elegantula* and *A. caerulea* (Ranunculaceae) in Colorado. *American Journal of Botany*, **65**, 406–414.
92. Miller, R.B. (1985) Hawkmoth pollination of *Aquilegia chrysantha* (Ranunculaceae) in southern Arizona. *The Southwestern Naturalist*, **30**, 69–76.
93. Moody-Weis, J.M. and Heywood, J.S. (2001) Pollination limitation to reproductive success in the Missouri evening primrose, *Oenothera macrocarpa* (Onagraceae). *American Journal of Botany*, **88**, 1615–1622.
94. Moré, M., Sérsic, A.N. & Cocucci, A.A. (2006) Specialized use of pollen vectors by *Caesalpinia gilliesii*, a legume species with brush-type flowers. *Biological Journal of the Linnean Society*, **88**, 579–592.
95. Moré, M., Sérsic, A.N. & Cocucci, A.A. (2007) Restriction of pollinator assemblage through flower length and width in three long-tongued hawkmoth-pollinated species of *Mandevilla* (Apocynaceae, Apocynoideae). *Annals of the Missouri Botanical Garden*, **94**, 485–504.
96. Morimoto, Y., Gikungu, M. and Maundu, P. (2004) Pollinators of the bottle gourd (*Lagenaria siceraria*) observed in Kenya. *International Journal of Tropical Insect Science*, **24**, 79–86.
97. Morinaga, S.I., Kumano, Y., Ota, A., Yamaoka, R. and Sakai, S. (2009) Day-night fluctuations in floral scent and their effects on reproductive success in *Lilium auratum*. *Population Ecology*, **51**, 187–195.
98. Neto, H.F.P. (2013) Floral biology and breeding system of *Bauhinia forficate* (Leguminosae: Caesalpinioideae), a moth-pollinated tree in southeastern Brazil. *Brazilian Journal of Botany*, **36**, 55–64.
99. Nilsson, L.A. (1983) Processes of isolation and introgressive interplay between *Platanthera bifolia* (l.) Rich and *P. chlorantha* (Custer) Reichb. (Orchidaceae). *Botanical Journal of the Linnean Society*, **87**, 325–350.
100. Nilsson, L.A. and Jonsson, L. (1985) The pollination specialization of *Habenaria decaryana* H. Perr. (Orchidaceae) in Madagascar. *Bulletin du Museum National d’Histoire Naturelle. Section B: Adansonia, Botanique, Phytochimie*, **7**, 161–166.
101. Nilsson, L.A. and Rabakonandrianina, E. (1988) Hawk-moth scale analysis and pollination specialization in the epilithic Malagasy endemic *Aerangis ellisii* (Reichenb. fil.) Schltr. (Orchidaceae). *Botanical Journal of the Linnean Society*, **97**, 49–61.
102. Nilsson, L.A., Jonsson, L., Rason, L. and Randianjohany, E. (1985) Monophily and pollination mechanisms in *Angraecum arachnites* Schltr. (Orchidaceae) in a guild of long-tongued hawk-moths (Sphingidae) in Madagascar. *Biological Journal of the Linnean Society*, **26**, 1–19.
103. Nilsson, L.A., Johnsson, L., Ralison, L. & Randrianjohany, E. (1987) Angraecoid orchids and hawkmoths in central Madagascar: specialized pollination systems and generalist foragers. *Biotropica*, **19**, 310–318.
104. Norman, J.K., Weller, S.G. and Sakai, A.K. (1997) Pollination biology and outcrossing rates in hermaphroditic *Schiedea lydgatei* (Caryophyllaceae). *American Journal of Botany*, **84**, 641–648.
105. Okamoto, T., Kawakita, A. & Kato, M. (2008) Floral adaptations to nocturnal moth pollination in *Diplomorpha* (Thymelaeaceae). *Plant Species Biology*, **23**, 192–201.
106. Oliveira, P.E. (1996) Floral biology of *Salvertia convallariodora* (Vochysiaceae): a hawk moth pollinated species of Brazilian cerrado vegetation. *Revista Brasileira de Botanica*, **19**, 49–53.
107. Oliveira, P.E. and Gibbs, P.E. (1994) Pollination biology and breeding systems of six *Vochysia* species (Vochysiaceae) in Central Brazil. *Journal of Tropical Ecology*, **10**, 509–522.
108. Oliveira, P.E., Gibbs, P.E. & Barbosa, A.A. (2004) Moth pollination of woody species in the Cerrados of Central Brazil: a case of so much owed to so few? *Plant Systematics and Evolution*, **245**, 41–54.
109. Ortega-Baes, P., Saravia, M., Suhring, S., Godinez-Alvarez, H. and Zamar, M. (2011) Reproductive biology of *Echinopsis terscheckii* (Cactaceae): the role of nocturnal and diurnal pollinators. *Plant Biology*, **13**, 33–40.
110. Patt, J.M., Merchant, M.W., Williams, D.R.E. & Meeuse, B.J.D. (1989) Pollination biology of *Platanthera stricta* (Orchidaceae) in Olympic National Park, Washington. *American Journal of Botany*, **76**, 1097–1106.
111. Pedron, M., Buzatto, C.R., Singer, R.B., Batista, J.A.N. and Moser, A. (2012) Pollination biology of four sympatric species of *Habenaria* (Orchidaeceae: Orchidinae) from southern Brazil. *Botanical Journal of the Linnean Society*, **170**, 141–156.
112. Pelletier, L., Brown, A., Otrysko, B. & McNeil, J.N. (2001) Entomophily of the cloudberry (*Rubus chamaemorus*). *Entomologia Experimentalis et Applicata*, **101**, 219–224.
113. Pellmyr, O., Thompson, J.N., Brown, J.M. & Harrison, R.G. (1996) Evolution of pollination and mutualism in the yucca moth lineage. *The American Naturalist*, **148**, 827–847.
114. Pérez-Barrales, R., Arroyo, J. and Armbruster, W.S. (2007) Differences in pollinator faunas may generate geographic differences in floral morphology and integration in *Narcissus papyraceus* (Amaryllidaceae). *Oikos*, **116**, 1904–1918.
115. Pérez-Nasser, N., Eguiarte, L.E. and Pinero, D. (1993) Mating system and genetic structure of the distylous tropical tree *Psychotria faxlucens* (Rubiaceae). *American Journal of Botany*, **80**, 45–52.
116. Perkins, G., Estes, J.R. and Thorp, R.W. (1975) Pollination of *Cnidoscolus texanus* (Euphorbiaceae) in south-central Oklahoma. *The Southwestern Naturalist*, **20**, 391–396.
117. Pettersson, M.W. (1991) Pollination by a guild of fluctuating moth populations: option for unspecialization in *Silene vulgaris*. *Journal of Ecology*, **79**, 591–604.
118. Piedade, L.H. and Ranga, N.T. (1993) The pollination ecology of *Galipea jasminiflora* Engler (Rutaceae). *Revista Brasileira de Botanica*, **16**, 151–157.
119. Piratelli, A.J., Pina-Rodrigues, F.C.M., Gandara, F.B., Santos, E.M.G. and Costa, L.G.S. (1998) Pollination biology of *Jacaratia spinosa* (Aubl) ADC. (Caricaceae) in a residual forest in southwest Brazil. *Revista Brasileira de Biologia*, **58**, 671–679.
120. Pool, A. (2008) A review of the genus *Pyrostegia* (Bignoniaceae). *Annals of the Missouri Botanical Garden*, **95**, 495–510.
121. Rodger, J.G., van Kleunen, M. and Johnson, S.D. (2010) Does specialized pollination impede plant invasions? *International Journal of Plant Sciences*, **171**, 382–391.
122. Saunders, N.E. and Sipes, S.D. (2006) Reproductive biology and pollination ecology of the rare Yellowstone Park endemic *Abronia ammophila* (Nyctaginaceae). *Plant Species Biology*, **21**, 75–84.
123. Schlumpberger, B.O., Cocucci, A.A., Moré, M., Sérsie, A.N. & Raguso, R.A. (2009) Extreme variation in floral characters and its consequences for pollinator attraction among populations of an Andean cactus. *Annals of Botany*, **103**, 1489–1500.
124. Sharma, S.B., Rana, A. and Chauhan, S.V.S. (2006) Reproductive biology of *Crataeva religiosa* Forst. *Current Science*, **90**, 716–720.
125. Silva, W.R. and Sazima, M. (1995) Hawkmoth pollination in *Cereus peruvianus*, a columnar cactus from southeastern Brazil. *Flora*, **190**, 339–343.
126. Silva-Montellano, A. and Eguiarte, L.E. (2003) Geographic patterns in the reproductive ecology of *Agave lechuguilla* (Agavaceae) in the Chihuahuan desert. I. Floral characteristics, visitors, and fecundity. *American Journal of Botany*, **90**, 377–387.
127. Singer, R.B. (2001) Pollination biology of *Habenaria parviflora* (Orchidaceae: Habenariinae) in southeastern Brazil. *Darwiniana*, **39**, 201–207.
128. Singer, R.B. (2002) The pollination biology of *Sauroglossum elatum* Lindl. (Orchidaceae: Spiranthinae): moth-pollination and protandry in neotropical Spiranthinae. *Botanical Journal of the Linnean Society*, **138**, 9–16.
129. Singer, R.B. and Cocucci, A.A. (1997) Eye attached hemipollinaria in the hawkmoth and settling moth pollination of *Habenaria* (Orchidaceae): a study on functional morphology in 5 species from subtropical South America. *Botanica Acta*, **110**, 328–337.
130. Singer, R.B. and Sazima, M. (2001) The pollination mechanism of three sympatric *Prescottia* (Orchidaceae: Prescottinae) species in Southeastern Brazil. *Annals of Botany*, **88**, 999–1005.
131. Smith, G.R. and Snow, G.E. (1976) Pollination ecology of *Platanthera* (*Habenaria*) *ciliaris* and *P. blephariglottis* (Orchidaceae). *Botanical Gazette*, **137**, 133–140.
132. Sreekala, A.K., Pandurangan, A.G., Ramasubbu, R. and Kulloli, S.K. (2008) Reproductive biology of *Impatiens coelotropis* Fischer, a critically endangered balsam from the Southern Western Ghats. *Current Science*, **95**, 386–388.
133. Sreekala, A.K., Pandurangan, A.G., Ramasubbu, R. and Kulloli, S.K. (2011) Pollination biology of *Impatiens cuspidata* Wight and Arn. (Balsaminaceae), a rare and endemic balsam of the Western Ghats, India. *Journal of Threatened Taxa*, **3**, 1818–1825.
134. Stephenson, A.G. and Thomas, W.W. (1977) Diurnal and nocturnal pollination of *Catalpa speciosa* (Bignoniaceae). *Systematic Botany*, **2**, 191–198.
135. Streisfeld, M.A. and Kohn, J.R. (2007) Environment and pollinator-mediated selection on parapatric floral races of *Mimulus aurantiacus. Journal of Evolutionary Biology*, **20**, 122–132.
136. Sugiura, S. and Yamazaki, K. (2005) Moth pollination of *Metaplexis japonica* (Apocynaceae): pollinaria transfer on the tip of the proboscis. *Journal of Plant Research*, **118**, 257–262.
137. Suzán, H., Nabhan, G.P. and Patten, D.T. (1994) Nurse plant and floral biology of a rare night-blooming cereus, *Peniocereus striatus* (Brandegee) F. Buxbaum. *Conservation Biology*, **8**, 461–470.
138. Tasen, W., Tangmitcharoen, S., Thakeaw, M. & Ogata, K. (2009) Insect pollination of *Aquilaria crassna* (Thymelaeaceae): effect of moths for the fruit setting in Thailand. *Journal of the Faculty of Agriculture, Kyushu University*, **54**, 321–328.
139. Thien, L.B., Bernhardt, P., Gibbs, G.W., Pellmyr, O., Bergström, G., Groth, I. and McPherson, G. (1985) The pollination of *Zygogynum* (Winteraceae) by a moth, *Sabatinca* (Micropterigidae): an ancient association? *Science*, **227**, 540–543.
140. Thompson, J.N. & Pellmyr, O. (1992) Mutualism with pollinating seed parasites amid co-pollinators: constraints on specialization. *Ecology*, **73**, 1780–1791.
141. Torres, C., Mimosa, M., Ferreira, M.F. & Galetto, L. (2013) Reproductive strategies of *Datura ferox*, an abundant invasive weed in agro-ecosystems from central Argentina. *Flora*, **208**, 253–258.
142. Valdivia, C.E. and Niemeyer, H.M. (2006) Do floral syndromes predict specialisation in plant pollination systems? Assessment of diurnal and nocturnal pollination of *Escallonia myrtoidea*. *New Zealand Journal of Botany*, **44**, 135–141.
143. Vogel, S. and Mueller-Doblies, D. (1975) A night blooming autumn *Narcissus* bulb structure and flower ecology of *Narcissus viridiflorus*. *Botanische Jahrbuecher fuer Systematik Pflanzengeschichte und Pflanzengeographie*, **96**, 427–447.
144. Voss, E.G. and Riefner, R.E. (1983) A pyralid moth (Lepidoptera) as pollinator of blunt-leaf orchid. *The Great Lakes Entomologist*, **16**, 57–60.
145. Westerbergh, A. (2004) An interaction between a specialized seed predator moth and its dioecious host plant shifting from parasitism to mutualism. *Oikos*, **105**, 564–574.
146. Westwood, A.R. & Borkowsky, C.L. (2004) Sphinx moth pollinators for the endangered western prairie fringed orchid, *Platanthera praeclara* in Manitoba, Canada. *Journal of the Lepidopterists’ Society*, **58**, 13–20.
147. Whigham, D.F. and McWethy, M. (1980) Studies on the pollination ecology of *Tipularia discolor* (Orchidaceae). *American Journal of Botany*, **67**, 550–555.
148. Wilkin, P. (1995) A new species of *Ipomoea* (Convolvulaceae) from Mexico State, Mexico, and its evolution. *Kew Bulletin*, **50**, 93–102.
149. Williamson, P.S., Muliani, L. and Janssen, G.K. (1994) Pollination biology of *Abronia macrocarpa* (Nyctaginaceae), an endangered Texas species. *Southwestern Naturalist*, **39**, 336–341.
150. Willmott, A.P. and Burquez, A. (1996) The pollination of *Merremia palmeri* (Convolvulaceae): can hawk moths be trusted? *American Journal of Botany*, **83**, 1050–1056.
151. Wolff, D., Witt, T., Jürgens, A. & Gottsberger, G. (2006) Nectar dynamics and reproductive success in *Saponaria officinalis* (Caryophyllaceae) in southern Germany. *Flora*, **201**, 353–364.
152. Yokota, S. and Yahara, T. (2012) Pollination biology of *Lilium japonicum* var. *abeanum* and var. *japonicum*: evidence of adaptation to the different availability of diurnal and nocturnal pollinators. *Plant Species Biology*, **27**, 96–105.
